# Fully automated multi-grid cryoEM screening using *Smart Leginon*

**DOI:** 10.1107/S2052252522010624

**Published:** 2023-01-01

**Authors:** Anchi Cheng, Paul T. Kim, Huihui Kuang, Joshua H. Mendez, Eugene Y. D. Chua, Kashyap Maruthi, Hui Wei, Anjelique Sawh, Mahira F. Aragon, Viacheslav Serbynovskyi, Kasahun Neselu, Edward T. Eng, Clinton S. Potter, Bridget Carragher, Tristan Bepler, Alex J. Noble

**Affiliations:** a Simons Electron Microscopy Center, New York Structural Biology Center, New York, NY, USA; b Simons Machine Learning Center, New York Structural Biology Center, New York, NY, USA; c Department of Biochemistry and Molecular Biophysics, Columbia University, New York, NY, USA; Boston University School of Medicine, USA

**Keywords:** cryo-electron microscopy, grid screening, automation, structural biology, computer vision, machine learning, single-particle cryoEM

## Abstract

Single-particle cryo-electron microscopy (cryoEM) is a swiftly growing method for understanding protein structure. With increasing demand for high-throughput, high-resolution cryoEM services comes greater demand for rapid and automated cryoEM grid and sample screening. During screening, optimal grids and sample conditions are identified for subsequent high-resolution data collection. Screening is a major bottleneck for new cryoEM projects because grids must be optimized for several factors, including grid type, grid hole size, sample concentration, buffer conditions, ice thickness and particle behavior. Even for mature projects, multiple grids are commonly screened to select a subset for high-resolution data collection. Here, machine learning and novel purpose-built image-processing and microscope-handling algorithms are incorporated into the automated data-collection software *Leginon*, to provide an open-source solution for fully automated high-throughput grid screening. This new version, broadly called *Smart Leginon*, emulates the actions of an operator in identifying areas on the grid to explore as potentially useful for data collection. *Smart Leginon Autoscreen* sequentially loads and examines grids from an automated specimen-exchange system to provide completely unattended grid screening across a set of grids. Comparisons between a multi-grid autoscreen session and conventional manual screening by 5 expert microscope operators are presented. On average, *Autoscreen* reduces operator time from ∼6 h to <10 min and provides a percentage of suitable images for evaluation comparable to the best operator. The ability of *Smart Leginon* to target holes that are particularly difficult to identify is analyzed. Finally, the utility of *Smart Leginon* is illustrated with three real-world multi-grid user screening/collection sessions, demonstrating the efficiency and flexibility of the software package. The fully automated functionality of *Smart Leginon* significantly reduces the burden on operator screening time, improves the throughput of screening and recovers idle microscope time, thereby improving availability of cryoEM services.

## Abstract 2

*Smart Leginon* leverages new machine-learning algorithms together with new microscope and image-handling algorithms to enable unattended, multi-grid cryoEM screening. *Smart Leginon Autoscreen* reduces microscope operator time from ∼6 h to <10 min for a typical session and recovers substantial idle microscope time outside of normal working hours.

## 1. Introduction

Over the past decade, single-particle cryo-electron microscopy (cryoEM) has become an established method for structure determination of macromolecular protein complexes ranging from ∼40 kDa to several megadaltons (Wu & Lander, 2020 ▸; Burton-Smith & Murata, 2021 ▸). A single-particle cryoEM project begins with the application of an aliquot of purified protein in solution to a holey foil substrate supported by a metal mesh, referred to as an EM grid. The bulk sample is then attenuated to a thin aqueous film, which is vitrified by plunging the grid into a cryogen (Chua *et al.*, 2022 ▸). The ideal outcome of this procedure is to have the proteins spread out as ‘single particles’ embedded in vitreous ice that is only slightly thicker than the largest diameter of the protein and at a concentration that enables the most efficient data collection (Taylor & Glaeser, 2008 ▸; Noble *et al.*, 2018 ▸). Producing suitable grids for high-resolution data collection almost always involves a series of optimization steps, with cryoEM screening required at each step to empirically examine the grids (Frank, 2006 ▸). Variables that can be optimized include grid mesh type (typically copper or gold), grid film substrate (typically carbon or gold), grid hole size, sample concentration, buffer conditions, ice thickness, additives (*e.g.* detergents) and particle behavior, *i.e.* preferred orientation and degradation (Chua *et al.*, 2022 ▸; Noble *et al.*, 2018 ▸; D’Imprima *et al.*, 2019 ▸). The effects of these variables on grid and sample quality require that the grids are to be examined in a cryo-transmission electron microscope (cryoTEM) at a series of magnifications, also called multi-scale imaging (MSI) (Cheng *et al.*, 2021 ▸), from a grid atlas composed of grid tile images to identify squares, to square magnification to identify regions inside squares, to hole magnification to identify holes in those regions, to exposure magnification to analyze protein behavior and quality (Fig. S1 of the supporting information). MSI screening allows the operator and researcher to estimate how many images of a quality suitable for high-resolution structure determination may be obtained from each grid.

Screening across these variables usually requires that significantly more grids are prepared and imaged than are used for a subsequent high-resolution data collection, particularly for new projects. Even for mature projects, poor reproducibility of grid quality typically requires that two to six or more grids are screened before settling on a small subset that is best for a long data collection on a high-end instrument. Current data-collection software packages available to the public [*e.g.* *Leginon* (Cheng *et al.*, 2021 ▸; Suloway *et al.*, 2005 ▸), *SerialEM* (Mastronarde, 2003 ▸), *UCSFImage4* (Li *et al.*, 2015 ▸), *TFS EPU* (Drulyte *et al.*, 2022 ▸; Deng *et al.*, 2021 ▸), *Gatan Latitude* and *JEOL JADAS* (Zhang *et al.*, 2009 ▸), and *AutoEMation* (Lei & Frank, 2005 ▸)] focus on exhausting the usable imaging area on a single grid. This is commonly achieved through a high degree of tuning of automated targeting parameters. The wide range of grid types, ice thicknesses and other confounding variables have prevented the development of a general, robust automated solution that performs as well as an expert human operator in multi-grid screening. As a result, the major time burden for a microscope operator is grid screening.

To address this problem, we have incorporated a machine-learning (ML) approach into our data-collection system *Leginon* (Cheng *et al.*, 2021 ▸; Suloway *et al.*, 2005 ▸), together with significant updates to the *Leginon* grid handling and image processing algorithms to provide a fully automated screening application with the goal of obtaining a set of images that can be used to assess overall grid quality and identify the best regions of each grid in the microscope. The ML and some of the computer vision algorithms described herein are part of the *Ptolemy* package which has been described in detail elsewhere (Kim *et al.*, 2023 ▸); the additional *Leginon* image processing algorithms are described in the Materials and methods[Sec sec2]. We broadly call this new version *Smart Leginon*. ‘Smart’ refers to our effort in reducing human intervention, where the incorporation of *Ptolemy* square and hole targeting for automated screening is our first step. *Smart Leginon* includes a simple command line workflow, called *Autoscreen*, that allows for an entire multi-grid screening session to be set up in <10 min and run fully unattended. Additionally, *Smart Leginon* functionality may be used as independent modules from within the existing *Leginon* graphical user interface (GUI). All software and algorithms described herein are open source, designed to be transferable to other collection software and to be extended with new functionality. *Leginon* is free and *Ptolemy* is protected by a license and is free for academic use.

We measured the performance of *Smart Leginon* in a variety of situations. First, *Smart Leginon Autoscreen* was used to screen 11 previously unseen mouse apoferritin (mApof) (Danev *et al.*, 2019 ▸) grids to assess the overall speed and robustness of the system compared with 5 expert human operators. To assess the outcomes, we measured the total screening time, total operator time, percentage of ‘good’ holes selected, ice thickness and CTF resolution estimates (herein, a ‘good’ hole is based on analysis at hole magnification, not exposure magnification). Next, we assessed the ability of *Smart Leginon* to successfully target on a wide range of grid types without adjusting any parameters, including gold and carbon substrates, multiple hole sizes and spacings, and a wide range of ice thicknesses and grid quality. Finally, we report on the application of *Smart Leginon* to three real-world multi-grid screening and collection sessions for users at our cryoEM facility.

After the initial public release of *Smart Leginon*, another fully automated cryoEM collection package that uses ML to interface with *SerialEM*, called *SmartScope* (Bouvette *et al.*, 2022 ▸), has been publicly released. Additionally, there are other pieces of software available that use ML to perform specific steps in the data-collection pipeline (Fan *et al.*, 2022 ▸; Yokoyama *et al.*, 2020 ▸, 2021 ▸).

## 2. Materials and methods

### 2.1. Implementation in *Leginon*

In order to integrate *Ptolemy* (Kim *et al.*, 2021 ▸) into *Leginon* and add the *Autoscreen* features, three additions were built into *Leginon*: (1) a workflow for running *Ptolemy* processes which returns segmentations, target coordinates, target scores and other metadata; (2) an algorithm to filter and sample the targets found by *Ptolemy*; and (3) a method to manage grid exchange and the MSI workflow for each grid. These modifications, described below, are available as of the *myami-3.6* release (http://leginon.org).

(1) Two new node classes were added to *Leginon* to utilize *Ptolemy*. MosaicScoreTargetFinder handles the lowest magnification images which executes *Ptolemy*’s lowmag_cli.py on each tile of the grid atlas (*i.e.* grid tile images of multiple squares; Fig. S1, upper-left image). Similarly, the ScoreTargetFinder node class handles hole magnification images (*i.e.* images of multiple holes; Fig. S1, bottom-left image), which executes *Ptolemy*’s medmag_cli.py and loads the results for processing. Each of these node classes then loads the full set of results from *Ptolemy* into *Leginon* in JSON format. Shell scripts are defined by the *Leginon* administrator to make *Ptolemy* calls so that it is possible to easily substitute *Ptolemy* with future versions. An additional step was added to merge together partial squares at the edges of adjacent tiles (Fig. S2). This extra merging step allows square targets to be evaluated on the full atlas image. The merged area becomes the sum of areas, the center of gravity becomes the merged target coordinate and the average mean intensity weighted by each target area gives the mean intensity of the merged square. The merged target also takes on the highest score of the targets it is merged from.

(2) Atlas grid tile images are filtered by considering only the squares within a defined square area range, while the filtering for hole magnification images includes the lower threshold of the *Ptolemy* score and the ice thickness filter as implemented in other *Leginon* TargetFinder node classes. For *Autoscreen* purposes, hole magnification filters are usually loosely set so only very bad selections (*e.g.* holes with cracks or with large ice crystal contamination) are eliminated. The hole magnification filters did not remove any potential targets in the mApof 11 grid screening comparison.

*Ptolemy* scores play their strongest role for the square finder for the atlas grid tile images. The area-filtered squares are put into *N*_g_ equally sized groups based on the chosen parameter, where square area is typically chosen as the parameter by which to separate groups in the results as presented herein. The highest-ranked (‘best’) squares from each group based on *Ptolemy* scores then creates a total of *N*_s_ squares to be targeted at higher magnifications. For example, if *N*_g_ = 4 and *N*_s_ = 8, then 2 squares from each group will be selected.

A sampling feature for the hole magnification image hole finder was added to the *Leginon* automated target finder base class. This presents the user with one additional setting to handle the sampling and to decide on the maximum number of targets *N*_h_ to include. Sampling in a given hole magnification image is produced by dividing the holes by a defined variable into *N*_h_ classes and then randomly sampling one instance in each class. Relative ice thickness, determined by average pixel intensity of a small group of pixels near the center of a hole, is used as the variable in this classification. The *Ptolemy* scores can also be used to filter the targets prior to sampling; however, this filter was not used for any of the results presented herein.

(3) The *Autoscreen* workflow is initiated by a command line python script that sets up session information and defines the task to perform in each session. The current options are ‘full MSI’, which performs unattended grid screening at all magnifications, and ‘atlas only’, which only collects an atlas for each grid. These required additional changes in the *Leginon* framework. Changes to the *Leginon* manager were made to switch sessions without disconnecting from instruments and to issue the grid exchange and workflow instruction to individual sessions when it is active. Settings were made to be recallable from a specified example session instead of the most recent session. Automated execution of the square finder was added as an option for the MosaicTargetFinder base class. A ‘Center between holes’ option was added to the current auto-creation of focus targets. This parameter-less algorithm analyzes the target lattice and places the focus target halfway between the lattice points nearest to the center of the hole magnification image, thus ensuring its maximal distance from any hole selections.

To fully realize unattended multi-grid screening with *Autoscreen*, we used *AutoIt* scripting (https://www.autoitscript.com) to emulate the GUI operations necessary to insert and retract the objective aperture in the TFS microscope API as these function calls are not available through the TFS microscope API. For our TFS Glacios microscope without an energy filter, we found that inserting the objective aperture improved both ice thickness estimations and contrast in high-magnification images. For all experiments herein, a 70 µm C2 aperture was used for all magnifications.

For the examples shown in this manuscript, *Ptolemy* was run on a single CPU core on the *Leginon* computer connected to the microscope, which was sufficient to keep up with collection in real time.

### 2.2. *Smart Leginon Autoscreen* workflow

**Figure 1:**
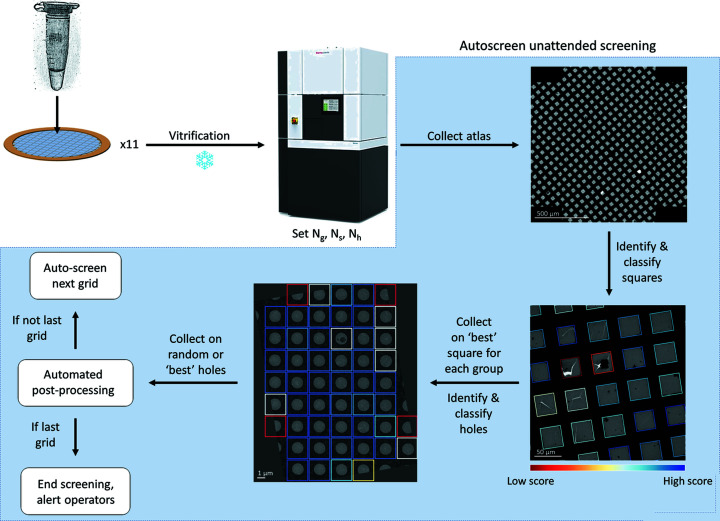
General *Smart Leginon Autoscreen* fully automated, unattended multi-grid screening workflow. CryoEM grids with samples are prepared and loaded into a cryoTEM with an automated specimen-exchange system. In *Autoscreen*, the microscope operator imports settings from an example session, including (1) *N*_g_, the number of groups to separate the squares in the grid atlas by, based on square area, mean intensity or *Ptolemy* score; (2) *N*_s_, the total number of squares that will be collected; and (3) *N*_h_, the number of holes that will be collected for each square. For each grid in the microscope, *Smart Leginon Autoscreen* will automatically target the highest-ranked (‘best’) square in each group and sample *N*_h_ random, threshold-filtered holes for each square, where *Ptolemy* determines rankings. Exposure magnification ice thickness estimations are determined in real time by *Leginon* and automated pre-processing in *Appion* (*e.g.* frame alignment and CTF estimation) is initiated at the end of each grid collection. If another grid is listed for screening, then *Autoscreen* automatically moves to the next grid and initiates a new collection session in the database. Once all grids are screened, *Autoscreen* safely returns the microscope to its default state and sends a message of completion to a designated Slack channel to alert operators and users. Each step in *Autoscreen* may be performed unattended by command line or used as independent modules in the *Leginon* GUI. Atlas scale bar is 500 µm, grid tile image scale bar is 50 µm, hole image scale bar is 1 µm.

Fig. 1 ▸ illustrates the general *Smart Leginon* workflow, with *Autoscreen* functionality highlighted in blue. The operator provides *Autoscreen* with a list of grids to be screened in the order in which they should be imaged and associates each grid with a specific project in the database. After the session is started, the following actions are performed unattended: (i) a grid is loaded from the automated specimen exchange system into the microscope; (ii) an atlas of the entire grid is collected tile by tile; (iii) *Ptolemy* locates all squares in the atlas tile images and merges the results (Kim *et al.*, 2021 ▸); (iv) *Leginon* separates squares into *N*_g_ groups based on a chosen parameter [currently either square area, mean intensity or *Ptolemy* score (Kim *et al.*, 2021 ▸)], and chooses the highest-ranked square from each group for a total of *N*_s_ squares; (v) *Leginon* acquires a square magnification image of each targeted square, then a raster of hole magnification images is acquired after the grid is set to eucentric height in the square; (vi) *Ptolemy* identifies the positions of holes within each hole magnification image (Kim *et al.*, 2021 ▸), (vii) Leginon selects *N*_h_ holes from this set based on chosen parameters (*e.g.* ice thickness, *Ptolemy* score, and/or random); (viii) *Leginon* performs a set of procedures to acquire an exposure magnification image from each hole, including focusing, setting defocus, checking for drift and normalizing lenses (Suloway *et al.*, 2005 ▸); (ix) once all squares and all holes from a grid have been imaged, the grid is unloaded from the stage. This process is then repeated until all grids have been examined, after which *Autoscreen* is terminated and a message is sent to the operators and users, typically through a designated SLACK channel (https://slack.com). During grid exchange, *Leginon* retracts and inserts the objective aperture automatically. *Leginon* performs ice thickness estimation (Cheng *et al.*, 2021 ▸; Rice *et al.*, 2018 ▸) for each exposure magnification image in real time. After each grid is screened, *Smart Leginon* initiates frame alignment and CTF estimation through *Appion* (Lander *et al.*, 2009 ▸). These image processing procedures are fast enough to be performed at the same rate as data collection. Exposure magnification collection targets may be augmented by collecting at several locations away from or in addition to the center of the holes identified by *Ptolemy*, *i.e.* multi-shot hole targeting (Suloway *et al.*, 2005 ▸). All of the images and the pre-processing results can be viewed in the *Appion* (Lander *et al.*, 2009 ▸) web-based three-way viewer (Figs. S1 and S3). Several components of the *Autoscreen* functionality may be used as independent modules from within the *Leginon* GUI, *e.g.* most hole finding at SEMC is now carried out using the *Ptolemy* hole finder.

### 2.3. Mouse apoferritin cryoEM grid preparation and screening

In total, 11 cryoEM grids were prepared by two people by adding 3 µl mouse apoferritin (mApof from the laboratory of Dr Kikkawa) solution (8 mg ml^−1^) to UltrAuFoil 1.2/1.3 grids (Quantifoil, Jena, Germany) immediately after plasma cleaning (Gatan Solarus II plasma cleaner; Gatan Inc. Pleasanton, CA, USA). The grids were blotted for 4 or 4.5 s, then vitrified by plunge-freezing in liquid ethane using a TFS Vitrobot Mark IV (Thermo Fisher Scientific) with the chamber maintained at 20°C and 100% humidity.

The 11 mApof cryoEM grids were screened on a TFS Glacios with a Falcon 3 camera (Thermo Fisher Scientific) in integration mode. The grids were not pre-screened prior to loading into the Glacios and starting *Smart Leginon*. Parameters used were *N*_g_ = 4, *N*_s_ = 4 and *N*_h_ = 5 where these and all other settings – including to split groups by square area and to rank squares in each group by *Ptolemy* score – were imported from a previous screening session that created an example *Smart Leginon* session (Fig. S4). Each atlas consisted of 22 grid tile images, where each tile was acquired at a magnification of 210× (2751 Å pixel^−1^). Square magnification was set to 940× (615 Å pixel^−1^); hole magnification was set to 5300× (109 Å pixel^−1^); exposure magnification was set to 120 000× (1.204 Å pixel^−1^). Exposure magnification movies were recorded in linear mode with a total exposure time of 400 ms across 40 frames and with an accumulated electron dose of 55.59 e^−^ Å^−2^ at −3 µm nominal defocus.

### 2.4. *Smart Leginon Autoscreen* versus operator quantification metrics

Timing measurements for *Smart Leginon Autoscreen* and human operators were obtained using the image timestamps at the beginning and end of each session. To reduce the bias of external microscope hardware, the time required for microscope alignment before the screening session and LN2 fillings were removed from the time measurements. Grid exchange times (∼5 min to retract a grid and insert the next grid) are included in the time measurements.

Hole quality analysis for mApof grids was performed visually. Only holes with more than ∼80% of the hole area existing inside the image (*i.e.* not significantly cut off by the edge of the image) were considered. A hole was considered contaminated if more than ∼40% of the imageable area in the hole was obfuscated.

### 2.5. Real-world *Smart Leginon Autoscreen* 35-grid user session

An assortment of grids – Quantifoil R1.2/1.3 300 mesh, Quantifoil R1.2/1.3 300 mesh with graphene, UltrAuFoil R1.2/1.3 300 mesh (Quantifoil, Jena, Germany) – were frozen with a TFS Vitrobot Mark IV (Thermo Fisher Scientific). In total, 35 user grids were screened on a TFS Glacios with a Falcon 3 camera in integration mode. *Autoscreen* settings were *N*_g_ = 3, *N*_s_ = 3 and *N*_h_ = 3. The imaging parameters were the same as the mApof screening session, except each atlas consisted of 43 grid tile images and the square magnification was set to 2600× (222 Å pixel^−1^).

### 2.6. Real-world *Smart Leginon* user sample screening and collection session

Eight user grids were screened on a TFS Glacios with a Falcon 3 camera in integration mode. *Autoscreen* settings were *N*_g_ = 4, *N*_s_ = 4 and *N*_h_ = 5. Imaging parameters were the same as the mApof screening session, except the atlases consisted of 28 grid tile images each. The two best grids were selected for a full data collection on a TFS Krios with a Gatan K3 camera in counting mode and BioQuantum energy filter (Gatan Inc. Pleasanton, CA, USA). Hole magnification was set to 3600× (97 Å pixel^−1^). Exposure magnification was set to 81000× (1.069 Å pixel^−1^). 2D classification and 3D refinement were performed with *CryoSparc* (version 3.3.1; Punjani *et al.*, 2017 ▸).

### 2.7. Real-world *Smart Leginon* user data-collection session

Grids were screened and collected on a TFS Krios with a Gatan K3 camera in counting mode, a BioQuantum energy filter and a Cs corrector. Hole magnification was set to 3600× (76 Å pixel^−1^). Exposure magnification was set to 81 000× (0.846 Å pixel^−1^).

### 2.8. Micrograph pre-processing

Motion correction was performed with *MotionCor2* (Zheng *et al.*, 2017 ▸) and CTF parameters of motion-corrected micrographs were estimated by *CTFFIND4* (Rohou & Grigorieff, 2015 ▸) through the *Appion* (Lander *et al.*, 2009 ▸) pipeline. Ice thickness was determined by the aperture limited scattering (ALS) method for Glacios sessions and by the energy filter method (Rice *et al.*, 2018 ▸) for Krios sessions from within *Leginon* (Cheng *et al.*, 2021 ▸); estimation by ALS is accurate to an estimated ±10 nm. For *Smart Leginon Autoscreen* sessions, AutoRelauncher.py was used to automatically re-launch the *Appion* real time pre-processing (*i.e.* frame alignment and CTF estimation) from an example session for many screening sessions described herein.

## 3. Results

### 3.1. *Smart Leginon Autoscreen* significantly decreases operator time while increasing microscope throughput

The *Smart Leginon Autoscreen* multi-grid screening performance using a TFS Glacios was evaluated by comparing it with 5 microscope operators who had not previously seen the grids nor the *Autoscreen* results. To obtain metrics on a per-grid basis, 11 grids were screened by *Autoscreen* in one *Smart Leginon* session to establish timing and performance values. Of these grids, 3 were selected for evaluation by 5 expert operators for a total of 15 operator grid screenings. *Autoscreen* and each operator targeted *N*_s_ = 4 squares across *N*_g_ = 4 groups and selected *N*_h_ = 5 holes per square. *Autoscreen* was set up to target holes randomly while operators selected squares manually and set up the standard *Leginon* template matching hole finder to target the 5 holes closest to the center of the image used for hole targeting; these are the standard methods that *Autoscreen* and operator use for screening grids. A variety of metrics were measured to assess the outcomes, including total screening time (*i.e.* the time from inserting the first grid into the microscope to the time the last grid is retracted), total operator time required during screening, the percentage of ‘good’ holes selected (*i.e.* non-empty, minimal contamination, no cracks), ice thickness estimation (Rice *et al.*, 2018 ▸) as measured from the exposure magnification images and CTF resolution estimation (Sheth *et al.*, 2015 ▸). Note (1) these hole and exposure magnification metrics do not conclusively determine whether an exposure image in a given hole will contain useful particles; however, these are some of the few metrics available for real time quality determination; and (2) random exposure targeting by *Autoscreen* versus targeting central holes by operators may result in systematic and random errors in ice thickness and CTF comparisons presented herein.

**Figure 2:**
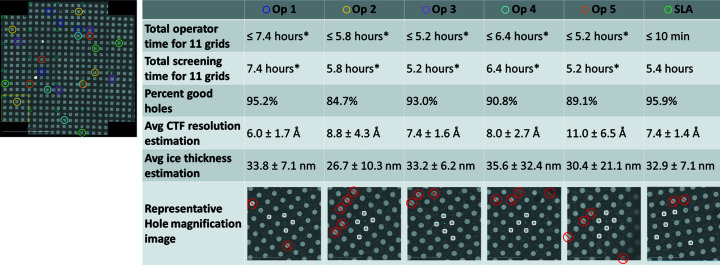
Quantitative comparisons between *Smart Leginon Autoscreen* (*SLA*) and expert microscope operators (Op). *Autoscreen* and operators each independently selected what they considered to be the ‘best’ 4 squares, where for *Autoscreen* each square was selected from 4 equally spaced square area ranges spanning all square areas (one grid atlas is shown on the left; see Fig. S8 for all atlases). *Autoscreen* took <10 min for the operator to set up and then run in a completely unattended manner for 5.4 h. The operators spend an average of 6.0 h to screen 11 grids (*extrapolated from 3 grids), of which most of the time is spent operating the microscope, interspersed with several short periods (5–10 min per grid) of time away from the microscope. (Note: calculations assume that the operators do not take any breaks away from the microscope.) In terms of percentage of good holes available from hole magnification images, *Autoscreen* (95.9% good holes) performed better than the average from the operators (90.6% good holes) and comparable to the best operator (95.2% good holes). Figs. S9–S14 show visual analyses of all holes and Table 1 ▸ shows the quantifications. From the random holes targeted (*SLA*) and central holes (Op), CTF resolution estimation for *Autoscreen* holes (7.3 ± 2.6 Å) was comparable to the average obtained by operators (7.4 ± 2.9 Å). Estimates of ice thickness show comparable values between *Autoscreen* (32.9 ± 7.1 nm) and operators (35.0 ± 18.7 nm). Table S1 ▸ shows the raw data. The hole magnification image most representative of the average in terms of hole quality is shown in the last row. Targets are shown by white squares and bad holes are shown by red circles with lines through them. Note: the representative image for operator 4 is a composite image due to there being no image that closely represents the average.

The *Autoscreen* collection sessions, including the example *Smart Leginon* session, took about 10 min for the operator to set up before beginning unattended collection. Screening of each grid then took an average of 29.7 ± 1.2 min to collect, resulting in about 5.4 h total to screen 11 grids. The equivalent tasks performed by 5 expert microscope operators on 3 of the 11 grids took 32.7 ± 7.1 min per grid, which extrapolates to 6.0 h to screen 11 grids if the operator rarely or never leaves the microscope. Although screening grids by an operator generally requires the operator to stay at the microscope during the entire process in order to perform several manual tasks, there does exist some time for the operator to non-optimally multitask. The amount of time depends strongly on the quality of the grids, which is unpredictable. During these tests, each operator did not interact with the microscope for 5–10 min of fragmented time per grid, which is generally not enough time to accomplish any meaningfully involved task. Thus, we estimate that the microscope operators have little to no meaningful time away from the microscope during their entire screening session. Fig. 2 ▸ summarizes the results.

The *Autoscreen* images at all magnifications were visually inspected in the web-based viewer that is part of the *Leginon* and *Appion* packages (Lander *et al.*, 2009 ▸) (Fig. S1) which quickly allowed the operator to determine that 8 of the grids were of good to excellent quality while the other 3 were of poor quality (Fig. S5). This analysis took about 5 min and allowed the operator to correctly correlate which grids were made by which person, exemplifying the efficiency of combining automated screening software with a database for storing data and a web GUI for rapid, remote visual analysis.

### 3.2. *Smart Leginon* square grouping and ranking enables *de novo* and prior-knowledge screening

One goal of cryoEM grid screening is to identify squares where optimal holes reside. For *de novo* cryoEM projects where no cryoEM screening has been performed, a common practice is to screen several different squares with different visible areas because the square area is often inversely proportional to the ice thickness of holes in the square (Fig. S6). On the other hand, if the sample owner has prior knowledge of the optimal square area for their sample, then the microscope operator will concentrate screening efforts on those squares.

*Smart Leginon* can be optimized for different stages of a project. Large values of square groups (*N*_g_) ensures that diversity is achieved in *de novo* cryoEM projects in a manner comparable with microscope operators. If the sample owner has prior knowledge of the sample behavior in ice, then the square area can be restricted to a range and *N*_g_ can be set to ‘1’ so that only the highest-ranked *N*_s_ squares within a specific area range are collected (Fig. S7).

Fig. S8 shows the 3 grids that were screened by *Smart Leginon Autoscreen* and independently by the 5 expert microscope operators. There is nearly no overlap (2.8% overlap) between the squares identified by *Autoscreen* and the operators, which is likely due to the fact that there are a large number of possible squares in each group area range and that several squares in each group are visually indistinguishable. Manual examination of each hole magnification image for *Autoscreen* and operator collections was performed to identify ‘bad’ holes (*i.e.* holes that have no ice, that have considerable contamination or that have cracks; Table 1 ▸, Figs. S9–S14). *Smart Leginon Autoscreen* using *Ptolemy* performed better at finding squares with good holes (95.9% good holes) compared with average operator performance (90.6% good holes).

This same level of performance was also evident when the exposure magnification images were analyzed. CTF resolution estimation of all exposure magnification images showed comparable performance between *Smart Leginon Autoscreen* (7.3 ± 2.6 Å) and operators (7.4 ± 2.9 Å). Ice thickness estimates for *Smart Leginon Autoscreen* (32.9 ± 7.1 nm) showed a comparable average and narrower range compared with the operators (35.0 ± 18.7 nm; Fig. 2 ▸, Table S1 ▸ of the supporting information).

### 3.3. *Smart Leginon* identifies holes and focus locations completely independent of grid type and hole size

**Figure 3:**
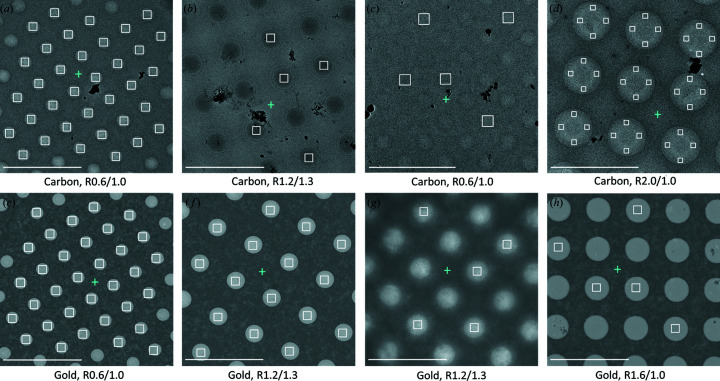
*Smart Leginon* hole finding using *Ptolemy* applied to Quantifoil grids of different materials, hole sizes, spacings and characteristics. The top row shows targeting on carbon foil grids and the bottom row on gold foil grids. (*a*) R0.6/1.0 carbon grid with minor contamination and uniform ice where all holes were identified and targeted (white field-of-view boxes). (*b*) R1.2/1.3 carbon grid with moderate contamination where the holes are darker than the surrounding foil, and all holes were identified and a random subset of 5 targeted. (*c*) R0.6/1.0 carbon grid with thick and non-uniform ice and minor contamination where all holes were identified and a random subset of 5 targeted. (*d*) R2.0/1.0 carbon grid with minor contamination where all holes were identified and targeted with 4 multi-shot targets. (*e*) R0.6/1.0 gold grid with minor contamination (top-left hole) where all holes were identified and all non-contaminated holes targeted. (*f*) R1.2/1.3 gold grid with no contamination where all holes were identified and targeted. (*g*) R1.2/1.3 gold grid with thick ice and no contamination where all holes were identified and a random subset of 5 targeted. (*h*) R1.6/1.0 gold grid with minor contamination where all holes were identified and a random subset of 5 targeted. Blue plus signs (+) are automatically determined focus locations. Note: an exclusion border was applied to each image, so some edge holes are not targeted. Scale bars are 5 µm.

To enable accurate and efficient automated exposure targeting, *Ptolemy* (Kim *et al.*, 2021 ▸) was integrated into *Smart Leginon* and two additional features were added: (1) a parameter-less algorithm that places the focus position halfway between the lattice that *Ptolemy* produces and closest to the center of the image, and (2) an exclusion border around the image to reduce the number of targets placed in partially cutoff holes. The *Smart Leginon* hole lattice and focus identification performance was tested on multiple different types of grids. We found that hole and focus-position finding for carbon film [Figs. 3 ▸(*a*)–3(*d*)] and gold film [Figs. 3 ▸(*e*)–3(*h*)] grids with varying hole sizes and spacings generally performs well without the need to adjust any parameters. Hole and focus targeting performed well under conditions where the template matching hole finder would have struggled or failed, for example on images where holes are darker than the surrounding film [Fig. 3 ▸(*b*)], on thick ice images with low contrast between the holes and foil [Fig. 3 ▸(*c*)], and on images with contamination [Figs. 3 ▸(*a*)–3(*d*)]. Additionally, multi-shot targeting may be set up in *Smart Leginon* [Fig. 3 ▸(*d*)] to maximize exposure area and target in particular locations across the gradient of the hole.

### 3.4. *Smart Leginon* and *Autoscreen* applied to a real-world 35-grid screening session

*Smart Leginon Autoscreen* was used to automatically screen 35 grids across 3 samples from one user on a TFS Glacios over 4 intensive days of concurrent grid optimization. Various grid types (carbon substrate, gold substrate and graphene-coated grids), sample concentrations and grid-making conditions (*i.e.* changes in blotting time on the Vitrobot) were attempted for each sample with the goal of preparing and identifying Krios-ready grids. Generally, grids were prepared during the day and screened automatically with *Autoscreen*, usually overnight, then the screening data were evaluated the next morning and used to guide the next iteration of grid and sample preparation. To prepare for screening all grids, the first grid was screened semi-manually while determining suitable *Smart Leginon* parameters to create an example session for *Autoscreen*. On two occasions, *Autoscreen* completed before the end of the working day, which allowed for preliminary data collection on the Glacios to be collected on the best grid overnight using *Smart Leginon* and the *Ptolemy* hole finder (Kim *et al.*, 2021 ▸). In total, 2594 micrographs were collected during one of these unattended overnight sessions resulting in a 5.6 Å structure, allowing for verification of the quality of the grid-making conditions for this sample. With an average automated screening time of ∼29 min per grid, *Autoscreen* and *Smart Leginon* enabled 35 grids to be automatically (for 34 grids) and semi-automatically (for the example grid) screened over ∼17 h of microscope time (overnight collection not included) during the 4-day period, allowing for a constant rapid feedback loop to the grid-making process. After all grids were prepared and screened, 12 grids were determined to be ready for Krios data collection. The *Smart Leginon* workflows allowed for the grid preparation and screening cycle to be significantly condensed, substantially increasing the time efficiency for the microscope, operator and researcher.

### 3.5. *Smart Leginon* applied to a real-world user sample screening and collection session

**Figure 4:**
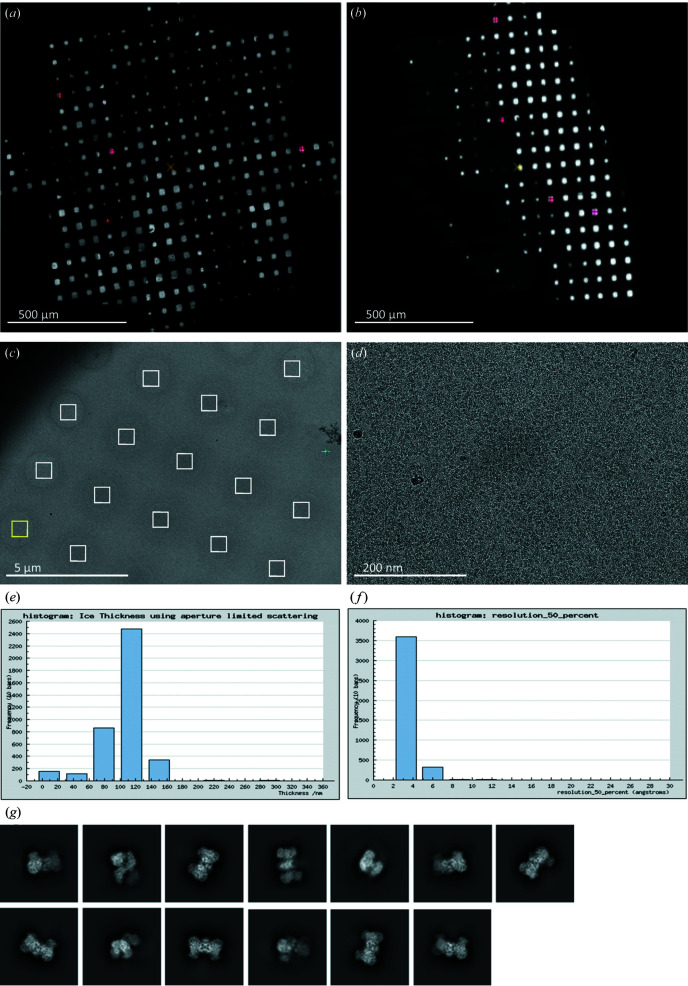
Real-world multi-grid screening with *Smart Leginon Autoscreen* followed by data collection with the *Smart Leginon* and the *Ptolemy* hole finder. *Autoscreen* readily allowed for the identification of (*a*) the best grid and (*b*) another decent grid. The *Ptolemy* hole finder from inside *Smart Leginon* was then used for high-quality data collection from (*c*) the best grid, leading to thousands of (*d*) exposure magnification images in (*e*) areas of moderately thick ice resulting in (*f*) relatively high-resolution CTF estimations, (*g*) high-quality 2D classes and a 3.1 Å structure (not shown).

*Smart Leginon Autoscreen* was used to *de novo* screen 8 user grids of an unspecified sample on a TFS Glacios. In general, all 8 grids had thick ice: 3 grids were completely opaque, 3 grids had a very limited number of good squares, and the remaining 2 grids had reasonable – though thick (≥100 nm) – ice and a sufficient number of good squares for high-resolution collection [Figs. 4 ▸(*a*) and 4(*b*)]. The best of these two grids was transferred to a TFS Krios for a full collection. During high-resolution data collection, the user chose to manually target squares, whereas the *Smart Leginon* implementation of *Ptolemy* was used for hole targeting (Kim *et al.*, 2021 ▸). The grid had a wide range of ice thicknesses: ice thickness variations resulted in some holes appearing lighter than the surrounding film [commonly observed, *e.g.* Fig. 3 ▸(*a*)], some holes appearing close to the same contrast as the surrounding film [Fig. 4 ▸(*c*)] and some appearing darker than the surrounding film [Fig. 3 ▸(*b*)]. *Ptolemy* within *Smart Leginon* allowed for holes in all cases to be identified reliably in an unattended manner without changing any parameters. The Krios session resulted in nearly 4000 exposure magnification images [Fig. 4 ▸(*d*)] whose ice thicknesses were primarily over 100 nm [Fig. 4 ▸(*e*)], yet reported good CTF resolution estimations [Fig. 4 ▸(*f*)], likely due to the highly concentrated proteins [Fig. 4 ▸(*d*)]. Subsequent 2D classification [Fig. 4 ▸(*g*)] and 3D refinement resulted in a 3.1 Å EM map (not shown here), which was sufficient for biological interpretation.

### 3.6. *Smart Leginon* screening applied to a real-world user data-collection session

A common user practice is to first screen sample and grid conditions on a screening microscope, then after variables are found that produce high-quality cryoEM grids, create several grids under the same conditions and load them directly into a high-end cryoTEM to avoid potential contamination during transfer from the screening microscope. However, the reproducibility of cryoEM grids under identical grid-making conditions is low. As a result, several grids often need to be screened in the high-end cryoTEM before ranking the grids for collection. This step again entails potentially hours of additional screening work by the microscope operator.

**Figure 5:**
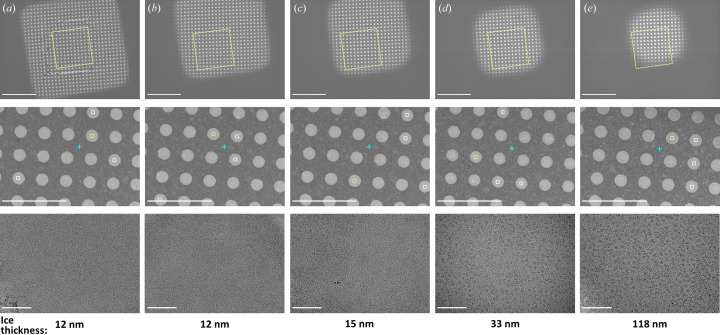
Real-world *Smart Leginon* screening MSI grid analysis of a user grid. The MSI analysis, with square magnification images of several squares from Grid 3 in Fig. S15 in the first row, hole magnification images of corresponding holes in the second row, exposure magnification images from inside corresponding holes in the third row, and ice thickness estimations in the fourth row, allowed the operator and user to determine that medium-sized squares contain 30–40 nm-thick ice containing (*d*) non-overlapping particles of interest, while (*a*)–(*c*) squares with ice thinner than 30 nm ice were void of particles and (*e*) squares with ice on the order of 100 nm thick had overlapping particles. Scale bars are 10 µm for the first row, 5 µm for the second row and 100 nm for the third row.

We employed *Smart Leginon* to screen 4 freshly made cryoEM grids on a TFS Krios prior to high-resolution data collection. The user had ordered the grids for data collection based on blot time according to previous screening results (Fig. S15). For each grid, *Ptolemy* square targeting (Kim *et al.*, 2021 ▸) together with *Smart Leginon* algorithms were used to select 5 squares across a range of square areas that were thought would be the most likely to contain well behaved particles. The operator then used *Smart Leginon* and the *Ptolemy* hole finder to automatically screen *N*_h_ = 4 holes per square in an unattended manner. After ∼30 min of screening for each grid, the operator and user deduced that (1) the optimal areas for collection were in squares with moderate ice thickness [30–40 nm; Fig. 5 ▸(*d*); red circles in Fig. S15] rather than thin ice areas [10–20 nm; Figs. 5 ▸(*a*)–5(*c*)] as anticipated, and (2) the anticipated grid ordering by the user was exactly the opposite of the optimal ordering as determined by *Smart Leginon* MSI analysis (Fig. S15). With this knowledge, the operator queued the best 2 grids using the *Ptolemy* hole finder in *Smart Leginon* for a 43 h collection session resulting in over 12 000 micrographs and a 2.6 Å EM map (not shown here). This use of *Smart Leginon* for 2 h of screening just prior to a high-resolution data collection proved critical for maximizing the efficiency of Krios time.

## 4. Discussion

Single-particle cryoEM throughput tracks X-ray crystallography throughput from 20 years ago (Berman *et al.*, 2000 ▸) and is poised for a throughput revolution (Drulyte *et al.*, 2022 ▸) much like the previous resolution revolution (Kühlbrandt, 2014 ▸). To keep up with the increasing demand from structural biologists, cryoEM developers must significantly reduce bottlenecks in the workflow. One significant bottleneck is the screening of cryoEM grids and samples prior to high-resolution collection. This step consumes a significant amount of user and microscope operator time that could be better used in other bottleneck areas such as sample preparation and data analysis. Fortunately, ML algorithms and image analysis have progressed to the point where most cryoEM screening tasks at the microscope can be performed without user intervention.

To directly address the cryoEM screening problem, we present *Smart Leginon* for fully automated grid screening. We illustrated the improvements of using *Smart Leginon Autoscreen* by experimentally testing the software against 5 expert microscope operators for 3 mApof grids and found that *Smart Leginon* required significantly less operator time at the microscope (<10 min for 11 grids compared with 6 h) while targeting comparable squares. We also showed that the *Ptolemy* parameter-less hole finder (Kim *et al.*, 2023 ▸) performed well on a range of common and difficult hole identification tasks with carbon and gold holey grids, including when contrast variation between the holes and the surrounding film reverses. The parameter-less hole finder suitably addresses the long-standing bottleneck of cryoEM hole identification. Lastly, we deployed *Smart Leginon* on 3 real-world multi-grid user samples to illustrate its utility: (1) a 4 day session where the user and operator iteratively screened 35 grids with *Autoscreen* and *Smart Leginon* while looping this rapid feedback into sample and grid optimization, allowing for 12 Krios-ready grids to be made; (2) a *de novo* cryoEM *Autoscreen* session of 8 grids where it was determined that relatively thick ice contained well behaved particles at reasonable concentration. The two best grids were collected using *Smart Leginon* and *Ptolemy*, resulting in a 3.1 Å EM map; and (3) a Krios session where 4 unseen grids were screened with *Smart Leginon* and *Ptolemy*, and the grid conditions were previously known and the grid order was prioritized by user based on identical grid preparation conditions. *Smart Leginon Autoscreen* results suggested better priority and the best two grids resulted in a 2.6 Å EM map. Additionally, *Smart Leginon Autoscreen* allowed for microscope idle time, including overnight and on weekends, to be used for screening owing to the minimal operator time required, further increasing the overall cryoEM throughput from sample preparation to high-resolution data collection.

The improvements in *Smart Leginon*, particularly the reduction of operator time from 6 h to <10 min for screening 11 grids, can be attributed equally to the ML algorithms and image processing in *Ptolemy* (Kim *et al.*, 2023 ▸) and the new purpose-built algorithms in *Leginon*. The targeting tasks have historically been performed by operators either manually or in a semi-automated manner where the operator either selects targets by hand or adjusts several parameters until the software reliably targets a narrow range of squares/holes. These specialized parameters, however, often do not translate well to new grids. Additionally, these semi-automated algorithms take time and expertise to use, and cannot account for wide variations in grid conditions, such as the grid in Figs. 3 ▸(*b*) and 4(*c*) which has alternating contrast between the holes and the surrounding film. In contrast, the *Smart Leginon* implementation of *Ptolemy* for hole finding has no parameters to change while square finding has only 4 parameters that need to be set at the outset of a multi-grid *Autoscreen* session: *N*_g_, *N*_s_, *N*_h_, and the range of either square area, mean intensity or *Ptolemy* score to search for squares within. Equally important, the ability of *Smart Leginon Autoscreen* to insert and retract grids and the objective aperture were critical for fully automating screening.

The *Smart Leginon* workflow (Fig. 1 ▸) may be extended in multiple ways, for instance: (1) the results from the *Smart Leginon* workflow may be augmented by numerous live-processing software packages (Punjani *et al.*, 2017 ▸; Kimanius *et al.*, 2021 ▸; Biyani *et al.*, 2017 ▸; Gómez-Blanco *et al.*, 2018 ▸; Stabrin *et al.*, 2020 ▸; Xie *et al.*, 2020 ▸; Caesar *et al.*, 2020 ▸; Tegunov & Cramer, 2019 ▸) that perform, for example, particle picking, *ab initio* model generation, 2D/3D classification and 3D refinement as automated post-processing routines; and (2) targeting may be further improved by feeding live-processing results back into the collection software as targeting priors.

### 4.1. Potential for implementation in other collection software

The image processing and ML routines from *Ptolemy* that were integrated together with the algorithms added into *Leginon* may also be integrated into other collection software packages, such as *SerialEM* (Mastronarde, 2003 ▸), *UCSFImage4* (Li *et al.*, 2015 ▸), *TFS EPU* (Deng *et al.*, 2021 ▸; Drulyte *et al.*, 2022 ▸), *Gatan Latitude*, *Jeol JADAS* (Zhang *et al.*, 2009 ▸) and *AutoEMation* (Lei & Frank, 2005 ▸). We describe in the Materials and Methods[Sec sec2] the exact modifications required to integrate *Ptolemy* into *Leginon* and to perform filtering and grid manipulation. The generalized requirements are: (1) a workflow for running *Ptolemy* processes and returning segmentations, target coordinates, target scores and other metadata that *Ptolemy* provides; (2) an algorithm to sample the targets found through automation; and (3) a method to manage grid exchange and the MSI workflow for each grid. Once these algorithmic requirements are met, then *Ptolemy* can be run in real time using a CPU on any modern computer with access to the same file system as the collection software. In addition to *Ptolemy* integration, several collection software modifications described in the Materials and Methods[Sec sec2] may need to be made, such as automatically controlling the objective aperture or integrating image processing routines for estimating clustered pixel intensities.

### 4.2. Using other ML hole and square target classifiers in *Smart Leginon Autoscreen*

There are a few hole and square target classifiers available (Yokoyama *et al.*, 2020 ▸; Yonekura *et al.*, 2021 ▸). These can be used in *Smart Leginon Autoscreen* as long as target finding results are provided by the classifier in the format accepted by the two ScoreTargetFinder node classes described here. For API details, please refer to https://emg.nysbc.org/redmine/projects/leginon/wiki/MSI-Ptolemy_API_information_for_developers

### 4.3. Current limitations of square selection

Due to the wide and sometimes unpredictable range of grid quality, grid characteristics and imaging characteristics, targeting can have issues. For instance, (1) we have found that large ice thickness gradients across individual atlas grid tiles can confuse the *Ptolemy* per-tile normalization (Kim *et al.*, 2023 ▸), causing square location predictions in seemingly random locations. (2) For grids where the majority of squares have cracks, *Ptolemy* may rank cracked squares higher than non-cracked squares. We hypothesize that the ML square ranking model becomes confounded because the brightness of a cracked square is greater than of a non-cracked square, yet still has other features of a non-cracked square, so it decides to weigh brightness highly. (3) For merged atlas tile images (Fig. S2), critical parameters such as square opening area may be distorted by any slight misalignment of the tiles, and the merged score, defined by the highest *Ptolemy* score, may not reflect the quality of the square if the image of the square had been intact in a tile. Solutions potentially include: adding a high-pass filtering step for (1), manually curating training data for (2) and increasing grid tile overlap for (3).

The grouping in the sampling algorithm used in the experiments described here aimed to form groups with equal numbers of scored squares. We noticed that this grouping approach made it difficult to achieve the diversity of square areas required for *de novo* screening when the distribution of square areas itself is highly unbalanced. For example, if most squares on the grids are dry, the few squares with ice all end up in the same sampling group and would only be sampled once whereas dry squares would be sampled multiple times. To address this, we added an option to use the area filter to place squares into a predefined area range. This eliminates the square area bias in sampling, but adds two extra user parameters that need to be defined by the grid mesh.

Using this new grouping, we have had some success in targeting squares on Spotiton (Wei *et al.*, 2018 ▸; Dandey *et al.*, 2020 ▸) and chameleon (Darrow *et al.*, 2021 ▸) nanowire grids which have characteristic stripes of sample across contiguous squares and no sample elsewhere on the grid. However, this approach is not reliable yet. We suspect that this is because the percentage of such grids in the *Ptolemy* training set was low (Kim *et al.*, 2023 ▸) and that usable squares do not usually have a significantly reduced area compared with the squares with no ice that make up the majority of squares. Work is in process to improve the success rate.

### 4.4. Current limitations of the hole selection

In our experimental design for the timing and performance comparison between *Autoscreen* versus manual screening (Fig. 2 ▸), no quality filtering was performed. In day to day operations, we have not found that these scores provided by *Ptolemy* (Kim *et al.*, 2023 ▸) are generally a better classifier than the ice thickness estimations. Additionally, the accuracy of the hole centers found by *Ptolemy* are compromised when holes are very different from the majority of the *Ptolemy* model training set. This includes cases when the hole magnification images are taken at higher magnification so that the lattice is less clearly defined (*e.g.* when there are 4 or less holes in the image). The square lattice-based hole selection implemented in *Ptolemy* is also not able to target tilted or highly bent grids (Fig. S16) or lacey grids. A solution for targeting on intentionally tilted grids may be to stretch the image in the direction orthogonal to the tilt axis, feed the stretched image to *Ptolemy* to find the hole lattice, then un-stretch the coordinates produced by *Ptolemy*.

We are working on addressing these square and hole selection limitations so that *Smart Leginon* and *Ptolemy* generalize to as many grid and imaging characteristics as possible.

## 5. Conclusions

We anticipate that *Smart Leginon Autoscreen* and associated functionality will significantly increase the throughput of cryoEM screening, an essential optimization step in the high-resolution single-particle cryoEM pipeline. Simultaneously, the smart target selection algorithms significantly reduce operator time spent at the microscope, thus allowing for more time to be dedicated towards grid/sample preparation and data analysis. Moreover, idle microscope time outside of business hours (*e.g.* overnight and weekends) may be recovered, leading to an even greater effective increase in throughput. We envision that algorithms will continue to improve, particularly in the direction of real time feedback from live processing, which may enable intelligent, fully automated high-resolution collection that replicates or surpasses human performance.

## 6. Software availability

*Smart Leginon* is freely and publicly available as two components: (1) *Leginon* and *Autoscreen* are in myami-3.6 release and above (http://leginon.org) and licensed under the Apache License, Version 2.0; and (2) *Ptolemy* is publicly available for academic use only (https://github.com/SMLC-NYSBC/ptolemy) and licensed under CC BY-NC 4.0. A tutorial for how to set up and use *Smart Leginon* and *Autoscreen* is available at https://emg.nysbc.org/redmine/projects/leginon/wiki/Multi-grid_autoscreening

## Supplementary Material

Supporting figures and tables. DOI: 10.1107/S2052252522010624/eh5015sup1.pdf

## Figures and Tables

**Table 1:** Hole analysis for each square image for the 3 mApof grids imaged by *Smart Leginon Autoscreen* (*SLA*) and the 5 expert microscope operators (and their average) Figs. S9–S14 show the annotated hole magnification images.

	Good	Empty	Contaminated	Cracked	Total	Percentage good (%)
Operator 1	495	3	7	15	520	95.2
Operator 2	431	62	9	7	509	84.7
Operator 3	479	22	3	11	515	93.0
Operator 4	454	21	8	17	500	90.8
Operator 5	451	49	5	1	506	89.1
Average						90.6
*SLA*	493	8	6	7	514	95.9
