# Supplementary material for: Fully automated multi-grid cryoEM screening using Smart Leginon
Source: IUCrJ. 2023 Jan 1;10(Pt 1):77–89. doi: 10.1107/S2052252522010624 (PMC9812217; doi:10.1107/S2052252522010624)
Supplement: Supplementary file 1 [file m-10-00077-sup1.pdf]

IUCrJ

Volume 10 (2023)

Supporting information for article:

Fully Automated Multi-Grid Cryo-EM Screening using Smart Leginon

Anchi Cheng, Paul Kim, Huihui Kuang, Joshua H. Mendez, Eugene Y. D. Chua, Kashyap Maruthi, Hui Wei, Anjelique Sawh, Mahira F. Aragon, Viacheslav Serbynovskyi, Kasahun Neselu, Edward T. Eng, Clinton S. Potter, Bridget Carragher, Tristan Bepler and Alex J. Noble

Supplemental Table 1: Exposure magnification CTF resolution and ice thickness comparisons

Operator 1		Operator 2		Operator 3		Operator 4		Operator 5		Smart Leginon	
CTF (Å)	Ice Thickness (nm)	CTF (Å)	Ice Thickness (nm)	CTF (Å)	Ice Thickness (nm)	CTF (Å)	Ice Thickness (nm)	CTF (Å)	Ice Thickness (nm)	CTF (Å)	Ice Thickness (nm)
**Grid 1**											
3.60	31.70	25.00	11.75	6.02	36.45	6.89	37.53	8.33	37.65	7.31	33.69
3.46	31.95	6.51	11.74	6.82	38.76	7.54	34.34	7.17	34.85	6.07	39.04
4.21	29.59	7.79	34.67	8.74	34.74	7.24	37.63	5.51	38.21	6.70	31.56
3.50	31.29	11.37	14.45	7.87	36.63	5.27	34.96	9.55	10.03	8.24	32.02
3.58	28.23	6.96	35.74	7.96	35.04	7.70	165.92	8.96	104.29	7.62	34.55
6.12	38.38	6.89	31.90	3.73	33.88	7.70	35.89	6.96	36.46	7.09	31.97
7.87	38.89	7.31	32.12	7.39	36.45	8.14	33.96	6.29	36.06	7.54	30.41
7.02	35.82	7.17	30.10	6.34	32.90	16.66	9.19	7.87	38.72	7.79	27.03
7.46	36.83	6.70	30.56	12.14	9.04	7.24	32.47	8.14	35.89	6.07	34.26
6.70	34.61	6.57	32.66	6.23	28.37	6.29	35.17	8.14	36.98	12.14	12.31
3.92	36.87	7.39	32.54	7.96	35.11	4.81	32.59	6.82	32.17	7.62	29.65
6.51	39.92	7.70	34.06	7.70	37.24	7.79	32.10	7.79	30.12	6.57	34.18
6.51	42.01	6.29	35.24	8.74	32.57	6.40	33.24	5.64	35.20	7.09	43.17
6.34	39.63	6.18	32.90	6.96	37.94	7.02	30.63	7.02	31.47	8.33	31.58
6.89	35.47	7.79	34.63	7.31	34.56	6.34	33.69	25.00	10.18	6.46	38.50
7.24	34.03	12.79	14.69	7.54	33.07	11.56	9.44	13.52	16.60	6.57	27.82

6.70	36.02	11.20	9.27	7.70	35.88	11.20	7.34	25.00	9.75	7.96	37.55
6.51	33.97	6.46	36.46	6.07	31.32	10.24	10.52	25.00	15.08	5.35	36.67
9.31	7.76	7.79	31.18	6.63	32.77	5.55	32.33	15.24	7.83	7.79	42.23
7.39	32.46	10.69	8.21	7.96	30.38	8.05	33.93	12.57	9.48	6.89	29.53
**Grid 2**											
6.70	37.52	5.92	27.02	7.31	30.83	6.96	28.52	6.29	35.59	6.70	28.21
6.76	38.78	8.33	26.37	4.29	29.71	4.87	31.20	7.79	39.37	8.05	28.96
8.96	18.78	7.87	24.39	6.82	26.72	7.02	26.76	8.53	32.82	7.31	25.86
13.03	18.21	6.82	25.74	7.17	28.11	6.02	29.06	5.55	33.85	9.19	29.76
8.74	12.03	8.74	24.36	5.78	27.01	5.83	27.88	8.33	33.61	25.00	-
7.54	33.90	6.18	32.53	6.12	23.96	6.57	26.67	5.87	32.90	4.75	28.27
7.24	31.73	7.46	32.91	6.76	25.25	7.31	28.98	6.57	33.50	4.91	27.40
5.19	30.43	6.70	31.34	6.70	22.92	6.76	23.81	6.23	30.81	5.55	24.11
9.55	30.36	5.92	33.05	7.09	23.52	5.47	24.39	7.96	32.14	5.97	25.86
5.97	26.92	5.69	32.59	7.54	21.77	6.29	23.85	6.76	30.78	6.02	30.07
7.31	26.68	5.69	27.74	7.62	21.34	7.02	27.30	11.56	9.27	6.34	26.05
3.50	29.95	5.64	30.38	6.63	21.91	7.46	28.02	7.46	30.66	5.27	30.61
3.56	25.85	7.24	24.64	12.57	17.62	5.97	24.46	8.96	20.82	7.87	24.96
6.51	27.32	5.78	25.12	13.78	27.27	7.70	26.01	8.33	40.03	6.70	37.78
7.02	28.65	6.82	24.45	25.00	12.37	10.24	7.82	8.43	22.96	6.89	28.18
7.96	31.38	7.24	29.19	6.96	33.05	7.46	32.34	6.46	31.23	6.12	29.53
5.64	31.08	5.69	30.93	7.79	34.01	7.39	35.29	7.24	33.89	7.79	26.84
3.56	26.66	6.70	28.28	6.57	30.59	10.24	22.65	9.07	30.55	7.87	33.70
8.24	29.44	6.76	28.82	6.07	31.56	7.24	33.07	7.31	31.50	7.96	23.42

3.79	27.20	6.29	28.21	7.46	34.15	7.54	33.39	7.46	35.28	8.24	25.55
**Grid 3**											
7.46	39.23	8.05	125.63	6.12	49.19	5.92	0.00	6.57	46.39	7.62	49.72
7.24	37.73	8.14	127.36	7.17	52.39	3.96	0.00	6.23	47.87	6.96	47.78
5.64	36.58	8.53	124.55	6.51	45.27	6.02	0.00	6.63	52.31	5.97	64.13
3.38	39.06	8.24	119.15	6.63	52.56	6.63	0.00	7.02	44.64	6.51	49.94
3.55	37.40	8.33	126.39	6.07	50.77	8.74	0.00	6.96	51.90	8.53	51.61
4.94	35.84	7.24	34.94	6.96	34.27	5.01	48.31	7.54	40.09	8.05	24.93
5.51	34.50	7.62	33.31	5.87	35.15	6.70	45.08	6.89	39.77	5.60	37.29
6.46	35.06	6.76	32.38	6.57	43.07	6.40	37.79	6.46	37.74	7.31	32.63
3.23	35.94	5.78	33.47	7.54	34.19	6.89	40.42	6.82	38.42	7.02	30.06
6.07	36.49	7.46	31.84	7.02	36.51	5.55	38.44	7.24	36.14	6.89	29.80
7.24	39.53	7.46	42.15	7.09	47.11	8.53	62.34	7.17	44.92	7.02	43.14
7.31	41.19	6.82	41.44	6.82	55.39	6.18	60.58	5.64	45.18	7.46	41.18
5.47	34.08	6.89	37.63	5.97	57.35	7.62	56.95	5.92	41.20	5.97	39.68
4.98	42.39	5.43	41.52	6.02	56.17	6.63	57.87	6.18	41.35	4.62	47.45
6.46	39.45	6.63	40.16	6.18	53.63	5.60	61.24	7.31	43.88	6.82	39.88
6.96	31.99	6.07	41.10	5.15	48.92	6.46	79.40	5.43	43.69	6.34	32.14
5.12	28.91	6.34	41.02	6.63	47.57	7.62	70.28	5.05	44.01	7.46	33.19
10.24	7.09	6.82	33.48	7.46	42.79	5.87	60.38	7.31	39.77	6.63	38.03
7.79	27.40	6.46	39.89	5.08	44.72	7.24	62.49	6.34	41.86	5.64	31.92
9.82	9.23	3.98	35.54	5.64	41.54	7.39	57.42	7.39	46.51	6.51	32.44
**Aggregated**											
**Grid 1**											

Avg CTF (Å)	6.04	Avg CTF (Å)	8.83	Avg CTF (Å)	7.39	Avg CTF (Å)	7.98	Avg CTF (Å)	11.03	Avg CTF (Å)	7.36
STDEV (Å)	1.71	STDEV (Å)	4.27	STDEV (Å)	1.59	STDEV (Å)	2.70	STDEV (Å)	6.53	STDEV (Å)	1.37
Avg Ice Thickness (nm)	33.77	Avg Ice Thickness (nm)	26.74	Avg Ice Thickness (nm)	33.16	Avg Ice Thickness (nm)	35.64	Avg Ice Thickness (nm)	30.35	Avg Ice Thickness (nm)	32.89
STDEV (nm)	7.11	STDEV (nm)	10.33	STDEV (nm)	6.24	STDEV (nm)	32.35	STDEV (nm)	21.09	STDEV (nm)	6.58
Min (nm)	7.76	Min (nm)	8.21	Min (nm)	9.04	Min (nm)	7.34	Min (nm)	7.83	Min (nm)	12.31
Max (nm)	42.01	Max (nm)	36.46	Max (nm)	38.76	Max (nm)	165.92	Max (nm)	104.29	Max (nm)	43.17
**Grid 2**											
Avg CTF (Å)	6.84	Avg CTF (Å)	6.67	Avg CTF (Å)	8.30	Avg CTF (Å)	7.07	Avg CTF (Å)	7.61	Avg CTF (Å)	7.72
STDEV (Å)	2.35	STDEV (Å)	0.92	STDEV (Å)	4.47	STDEV (Å)	1.33	STDEV (Å)	1.39	STDEV (Å)	4.24
Avg Ice Thickness (nm)	28.14	Avg Ice Thickness (nm)	28.40	Avg Ice Thickness (nm)	26.18	Avg Ice Thickness (nm)	27.07	Avg Ice Thickness (nm)	31.08	Avg Ice Thickness (nm)	28.16
STDEV (nm)	6.24	STDEV (nm)	3.10	STDEV (nm)	5.66	STDEV (nm)	5.74	STDEV (nm)	6.77	STDEV (nm)	3.43
Min (nm)	12.03	Min (nm)	24.36	Min (nm)	12.37	Min (nm)	7.82	Min (nm)	9.27	Min (nm)	23.42
Max (nm)	38.78	Max (nm)	33.05	Max (nm)	34.15	Max (nm)	35.29	Max (nm)	40.03	Max (nm)	37.78
**Grid 3**											
Avg CTF (Å)	6.24	Avg CTF (Å)	6.95	Avg CTF (Å)	6.43	Avg CTF (Å)	6.55	Avg CTF (Å)	6.60	Avg CTF (Å)	6.75
STDEV (Å)	1.88	STDEV (Å)	1.12	STDEV (Å)	0.70	STDEV (Å)	1.14	STDEV (Å)	0.70	STDEV (Å)	0.91

Avg Ice Thickness (nm)	33.45	Avg Ice Thickness (nm)	59.15	Avg Ice Thickness (nm)	46.43	Avg Ice Thickness (nm)	41.95	Avg Ice Thickness (nm)	43.38	Avg Ice Thickness (nm)	39.85
STDEV (nm)	9.42	STDEV (nm)	38.95	STDEV (nm)	7.36	STDEV (nm)	26.85	STDEV (nm)	4.33	STDEV (nm)	9.64
Min (nm)	7.09	Min (nm)	31.84	Min (nm)	34.19	Min (nm)	0.00	Min (nm)	36.14	Min (nm)	24.93
Max (nm)	42.39	Max (nm)	127.36	Max (nm)	57.35	Max (nm)	79.40	Max (nm)	52.31	Max (nm)	64.13
**All Grids Overall**											
Avg CTF (Å)	6.37	Avg CTF (Å)	7.48	Avg CTF (Å)	7.37	Avg CTF (Å)	7.20	Avg CTF (Å)	8.41	Avg CTF (Å)	7.28
STDEV (Å)	1.99	STDEV (Å)	2.73	STDEV (Å)	2.83	STDEV (Å)	1.92	STDEV (Å)	4.26	STDEV (Å)	2.61
Avg Ice Thickness (nm)	31.79	Avg Ice Thickness (nm)	38.10	Avg Ice Thickness (nm)	35.26	Avg Ice Thickness (nm)	34.89	Avg Ice Thickness (nm)	34.94	Avg Ice Thickness (nm)	33.73
STDEV (nm)	8.01	STDEV (nm)	27.42	STDEV (nm)	10.58	STDEV (nm)	24.85	STDEV (nm)	14.16	STDEV (nm)	8.46
Min (nm)	7.09	Min (nm)	8.21	Min (nm)	9.04	Min (nm)	0.00	Min (nm)	7.83	Min (nm)	12.31
Max (nm)	42.39	Max (nm)	127.36	Max (nm)	57.35	Max (nm)	165.92	Max (nm)	104.29	Max (nm)	64.13

**All Grids and Operators Overall**	
Avg CTF (Å)	7.37
STDEV (Å)	2.93
Avg Ice Thickness (nm)	34.99

STDEV (nm)	18.67
Min (nm)	0.00
Max (nm)	165.92

Supplemental Table 1 | CTF resolution and ice thickness estimates along with averages and standard deviations for Exposure magnification images from the three mApof grids that were screened by Smart Leginon Autoscreen and the five microscope operators. Negative ice thickness estimates are reported as 0.00 nm. Note: Ice thickness calibration on the Glacios has an estimated error of about ±10 nm, so the values shown here should be considered as relative values, not absolute.

Figure S1: Multi-scale imaging (MSI) visualization with the Appion 3 Way Viewer

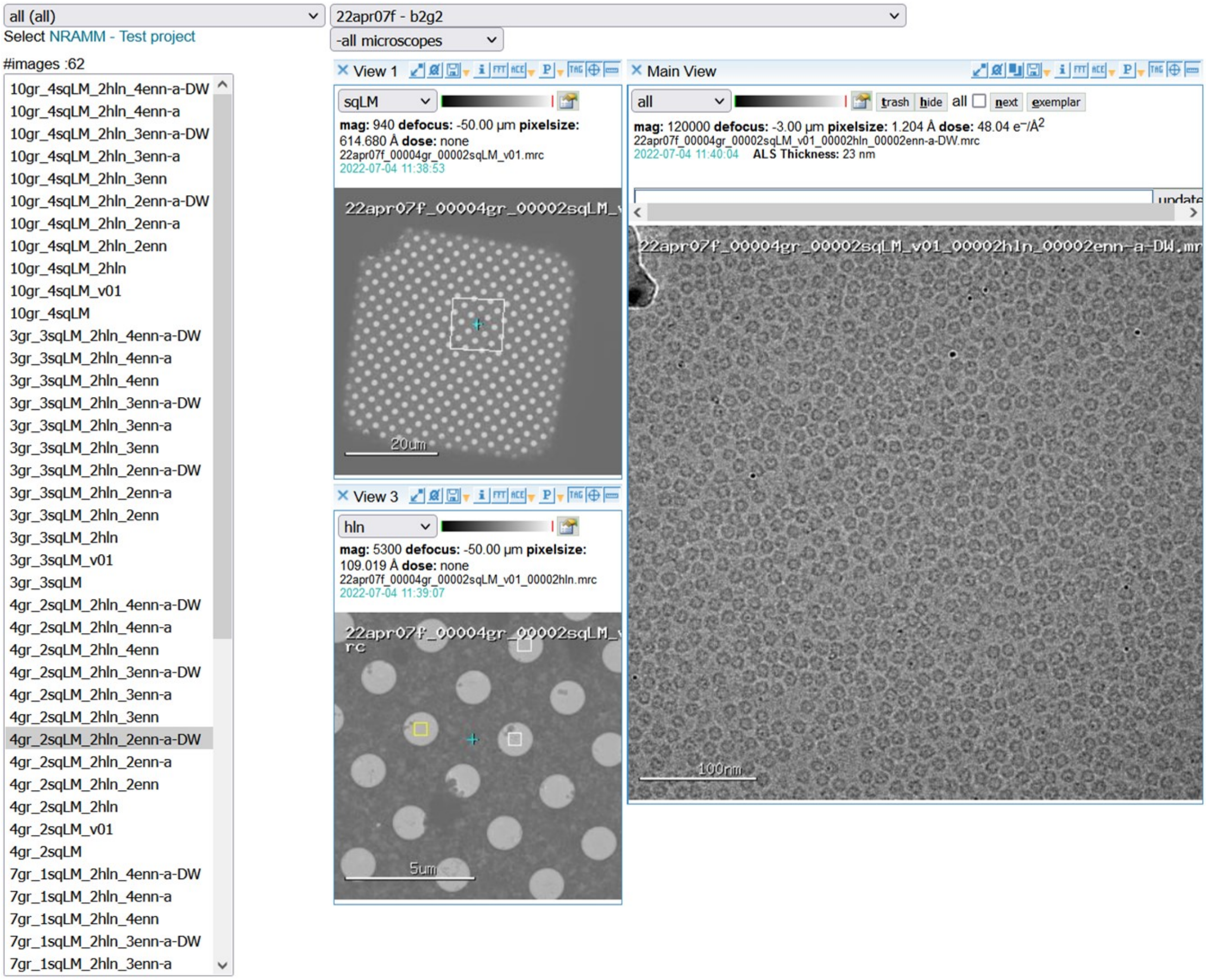

Figure S1 | The Appion 3 Way Viewer allows for rapid visualization of a full set of MSI images; the atlas (not shown), the Square magnification image (top-left), the corresponding Hole magnification image (bottom-left), and the corresponding Exposure magnification image (right).

Figure S2: Smart Leginon atlas grid tile image merging

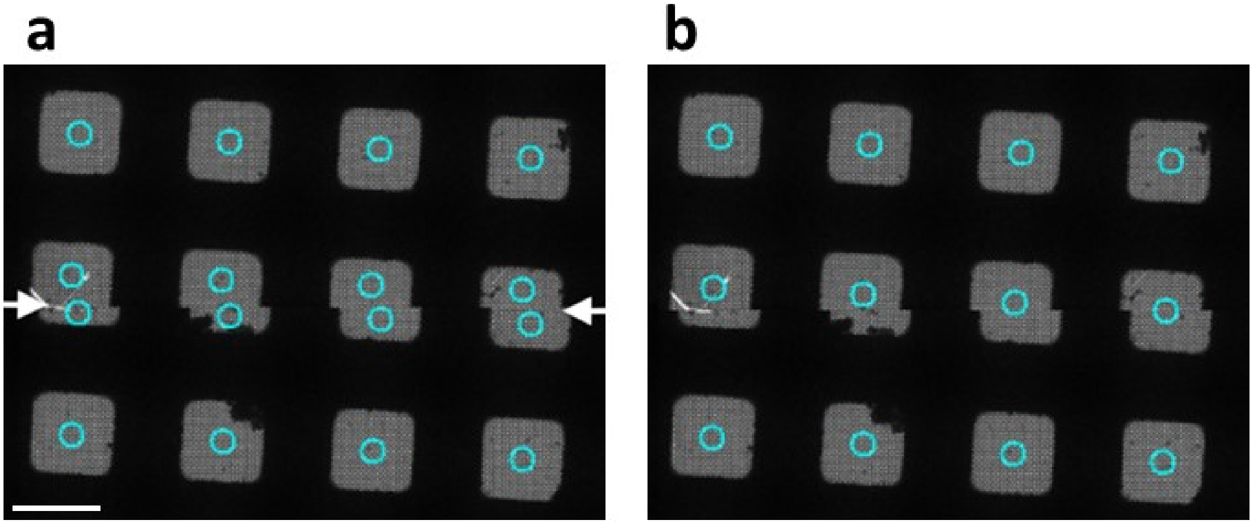

Figure S2 | The atlas grid tile image merging update to Leginon. (a) Two tiles - top and bottom halves of the image - where Ptolemy returned square locations and metadata for each image. The squares at the edge of the tiles end up with two sets of square locations and metadata. (b) The tiles after square location and metadata merging. Scale bar is 50 µm.

Figure S3: Appion 3 Way Viewer for analyzing pre-processing results

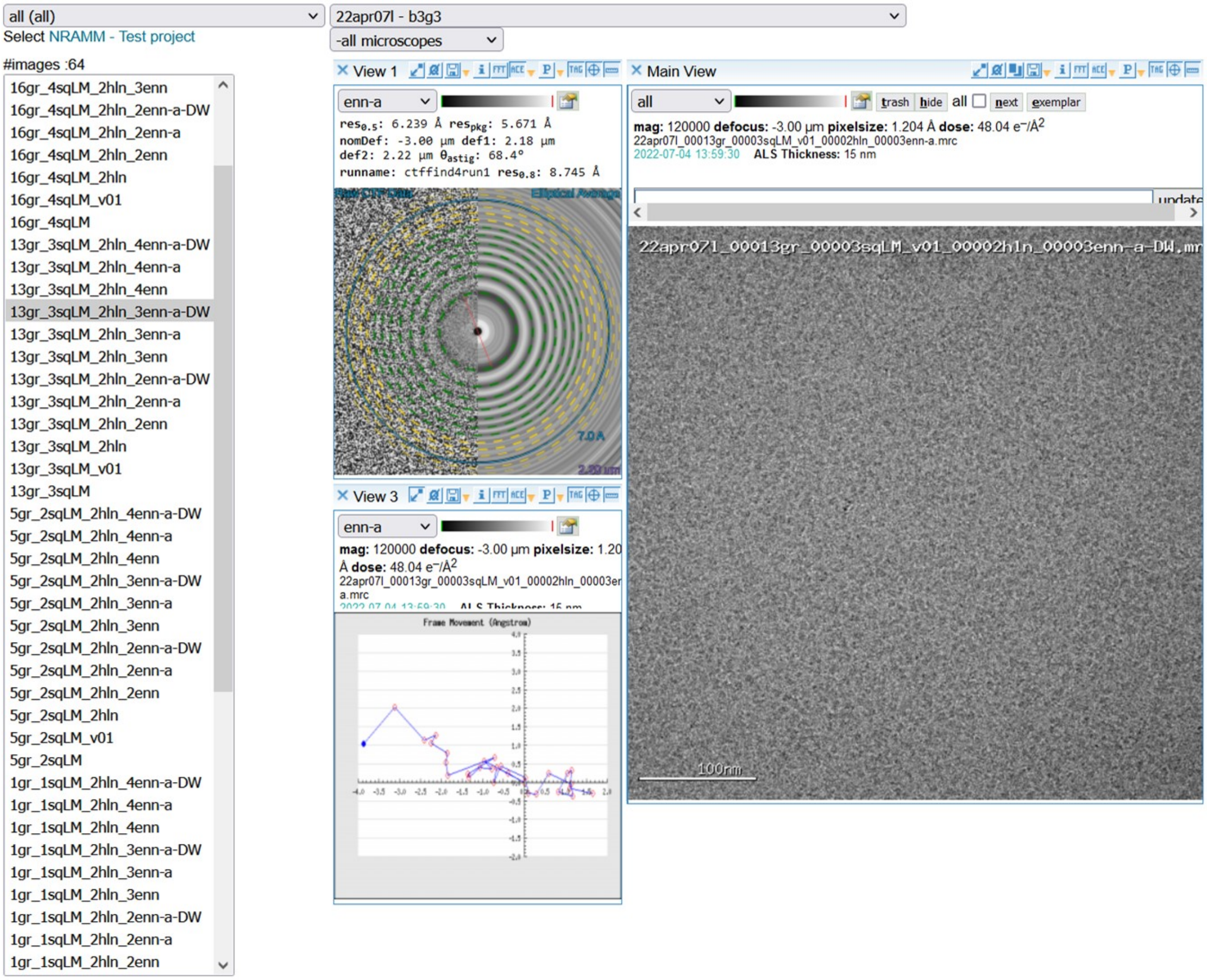

Figure S3 | The Appion 3 Way Viewer allows for near real-time analysis of Exposure magnification micrograph quality, including ice thickness estimation, CTF estimation, and frame alignment results, as shown here.

Figure S4: Smart Leginon Autoscreen command line workflow

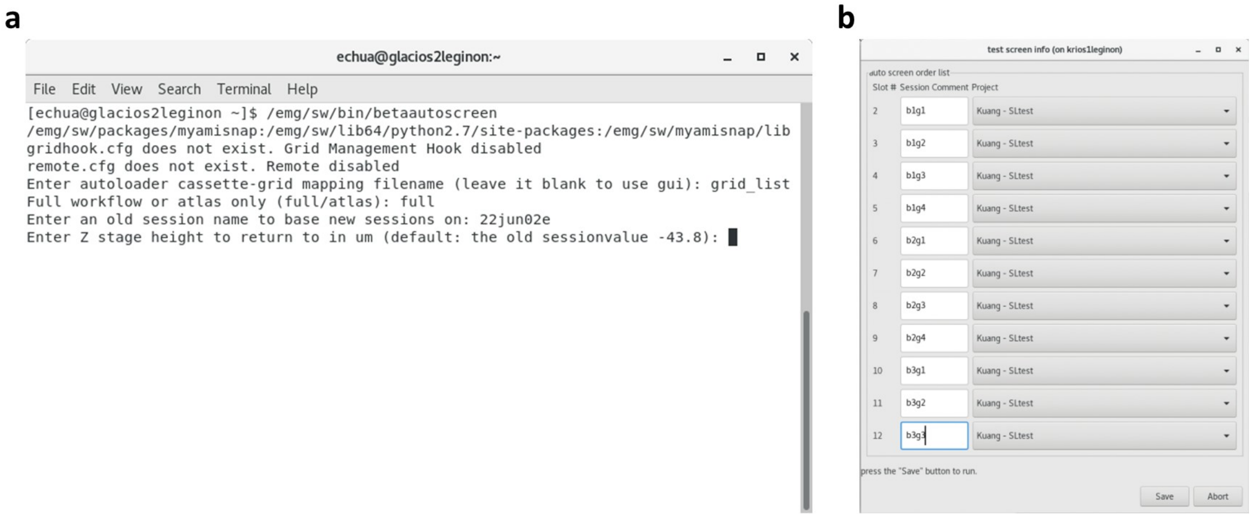

Figure S4 | Smart Leginon Autoscreen is executed by command line where the operator is asked to enter four pieces of information (a), then Autoscreen will screen all grids in the microscope completely unattended. The first piece of information is the names of each grid and corresponding projects that Leginon will associate them with; names can be listed in a file and the filename can be entered as in (a), or the entry can be left blank to open a GUI (b). Next, the operator selects whether to perform the full MSI workflow on all grids, or to only collect atlases of all grids. Next, the operator inputs the name of an example Smart Leginon session from which all settings will be imported for screening all grids. Lastly, the default stage Z height may be changed, if necessary.

Figure S5: Atlases collected by Smart Leginon Autoscreen

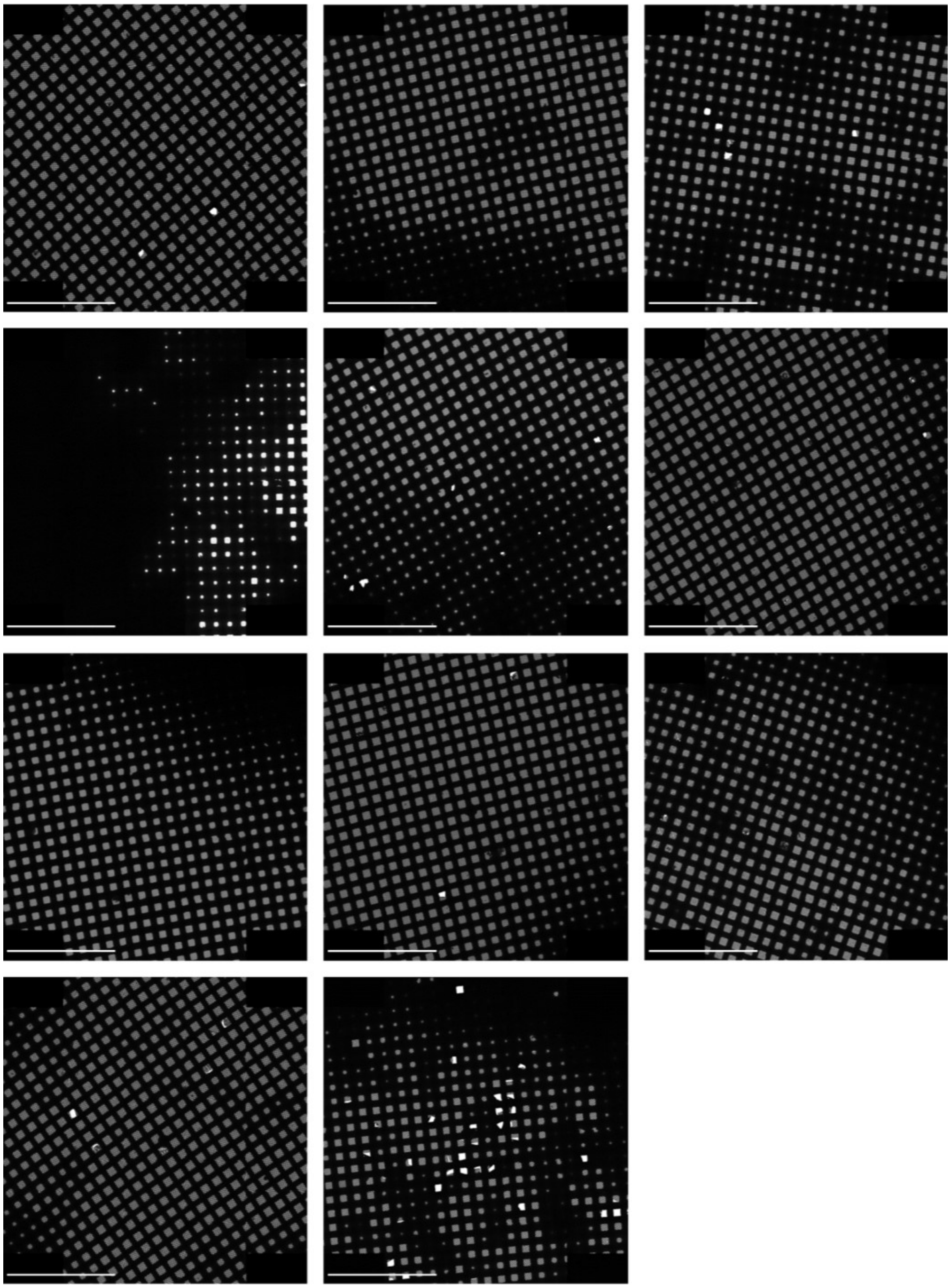

Figure S5 | Atlas images of 11 grids after Smart Leginon Autoscreen allowed for the rapid identification of 8 good to excellent grids (made by one person) and 3 poor grids (made by another). Poor grids are: First row, third column; second row, first column; and fourth row, second column. Scale bars are 500 µm.

Figure S6: Square area correlates with ice thickness

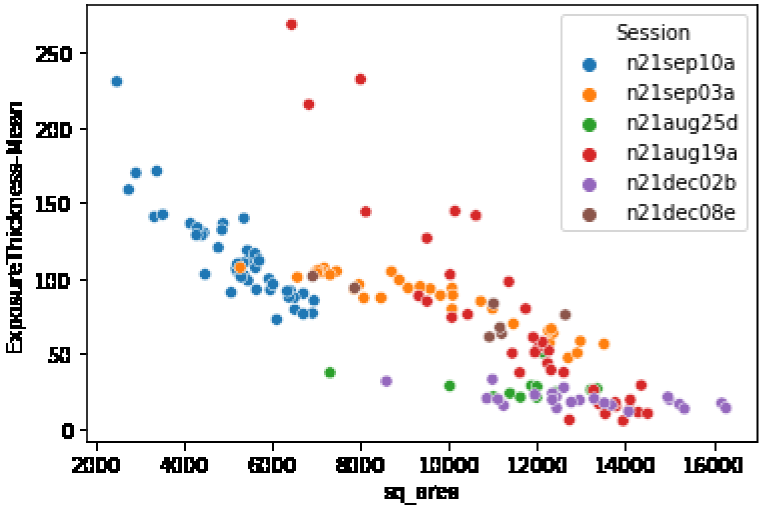

Figure S6 | Average ice thickness (nm) of holes in squares estimated from Exposure magnification images using an energy filter for calibration (y-axis) versus square area (arbitrary units) as calculated by Ptolemy (x-axis) across several collection sessions of different grids. In general, the larger the square area, the thinner the ice.

Figure S7: Smart Leginon can screen squares of a particular area

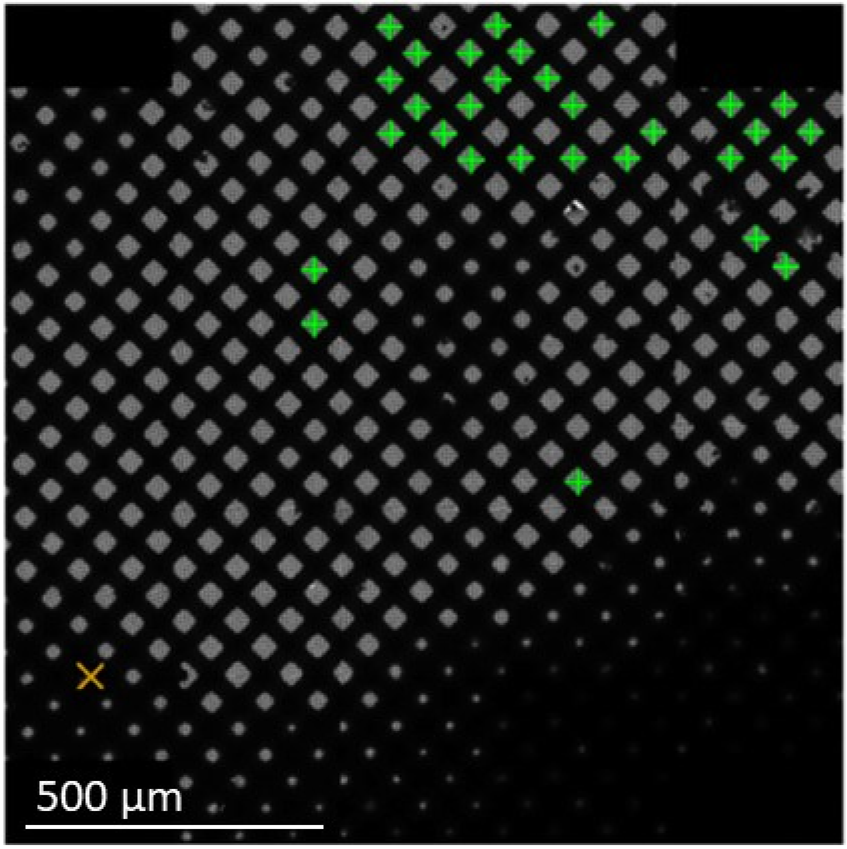

Figure S7 | Smart Leginon may be used to screen squares in a particular area range. In this example, the user has prior knowledge that their particles behave well in large squares which correspond to thin ice, thus Smart Leginon was set up to screen the 30 highest-ranked large squares (green plus symbols, +). (Note: The orange X symbol is the current location of the stage.)

Figure S8: Comparison between Smart Leginon Autoscreen and Operators’ independent square targeting

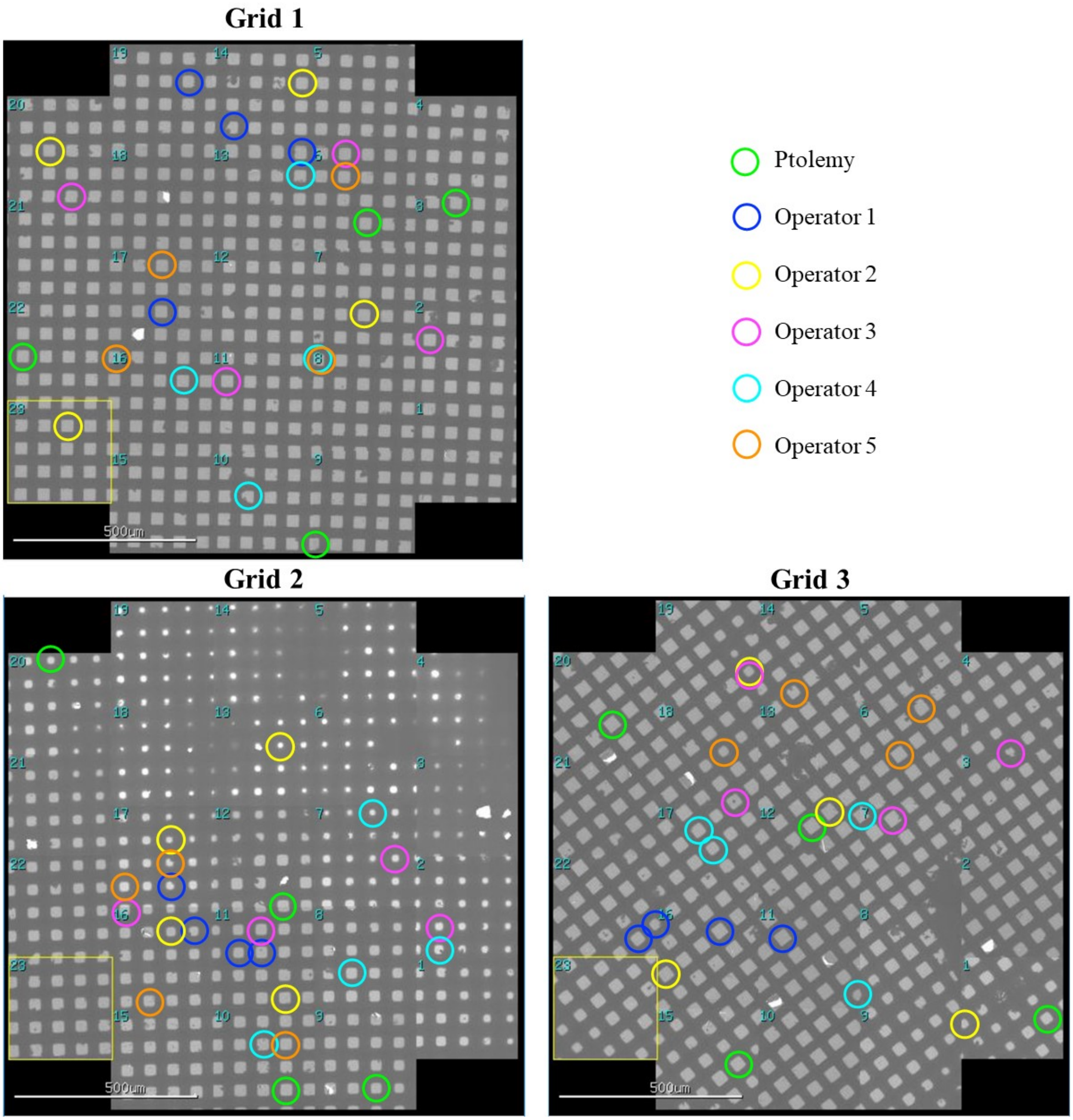

Figure S8 | A comparison of three unseen grids between Smart Leginon targeting and five expert microscope operators’ independent targeting. For each grid, Smart Leginon and the operators were instructed to choose the ‘best’ square from four equal-sized groups of square areas. Scale bars are 500 µm.

Figure S9: Analysis of holes from each square image for Operator 1

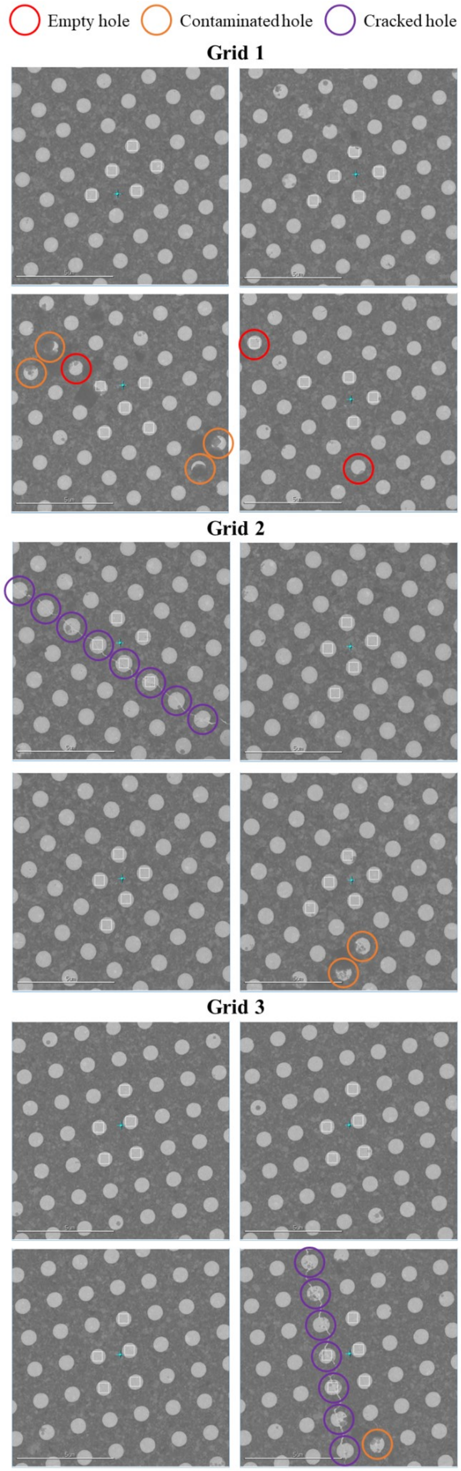

Figure S9 | Empty, contaminated, and cracked holes are identified for four squares from three grids collected by operator 1. The remaining holes are considered 'good'. Scale bars are 5 µm. (Note: White squares in Supplemental Figures 9-14 are exposure locations.)

Figure S10: Analysis of holes from each square image for Operator 2

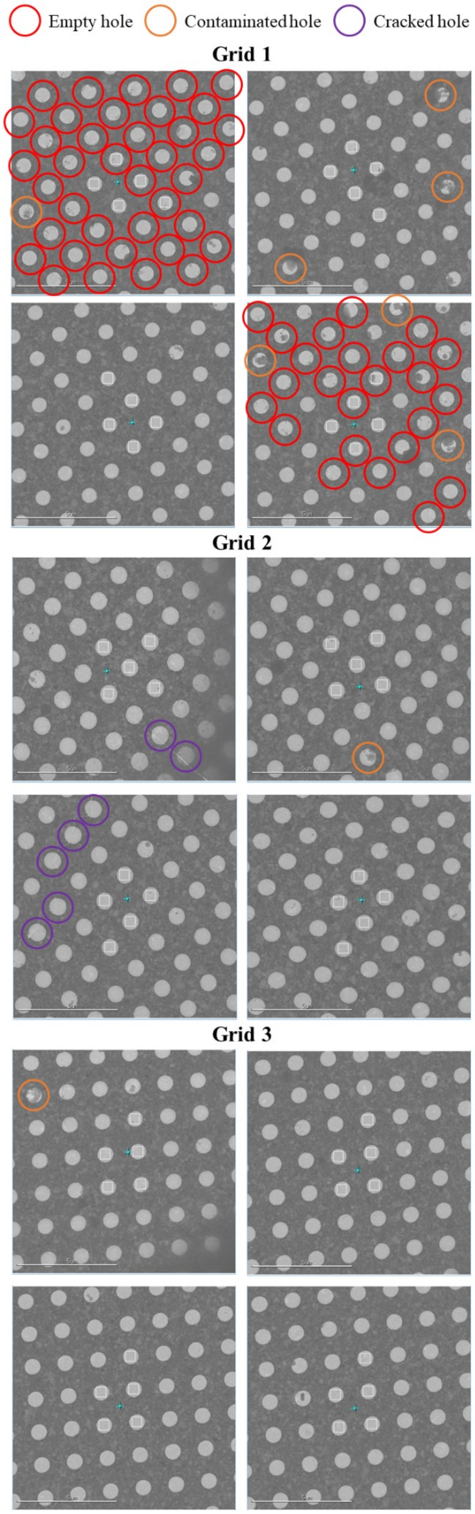

Figure S10 | Empty, contaminated, and cracked holes are identified for four squares from three grids collected by operator 2. The remaining holes are considered 'good'. Scale bars are 5 µm.

Figure S11: Analysis of holes from each square image for Operator 3

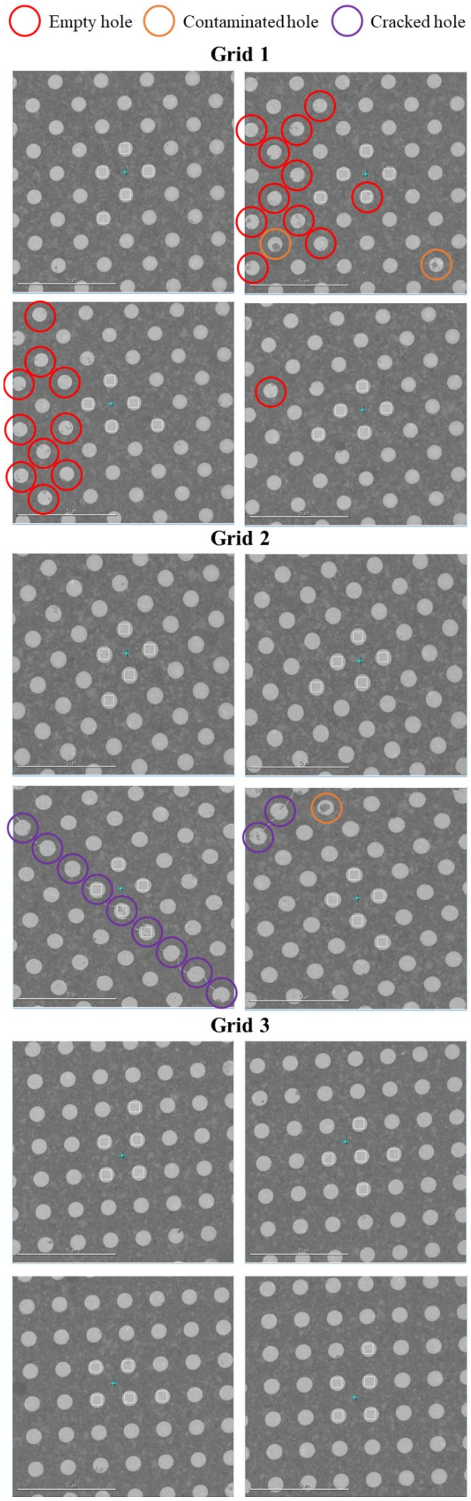

Figure S11 | Empty, contaminated, and cracked holes are identified for four squares from three grids collected by operator 3. The remaining holes are considered 'good'. Scale bars are 5 µm.

Figure S12: Analysis of holes from each square image for Operator 4

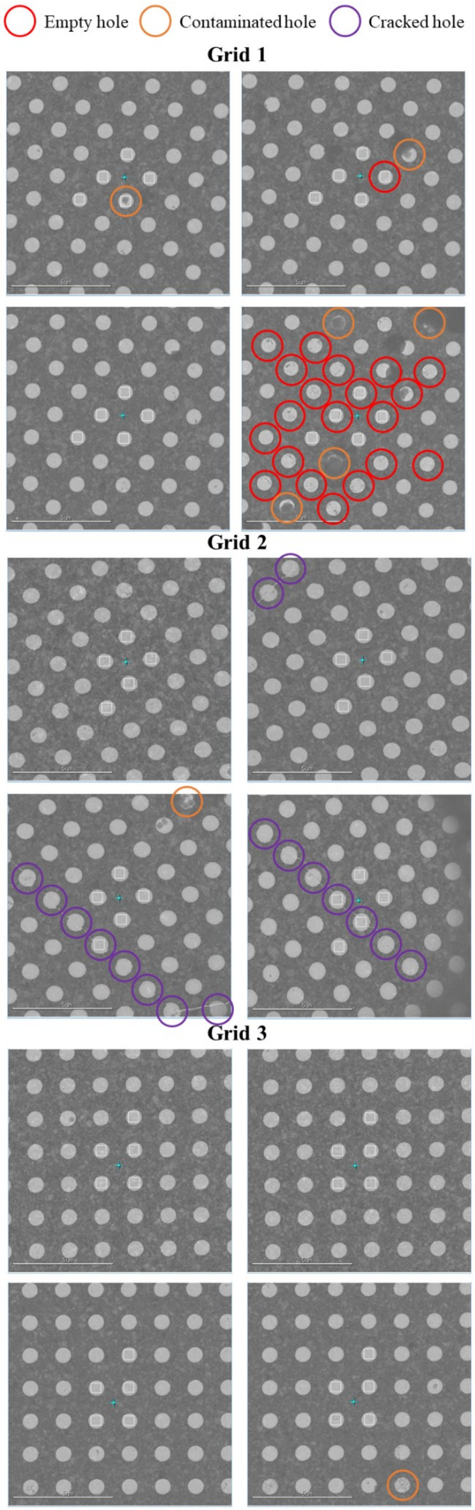

Figure S12 | Empty, contaminated, and cracked holes are identified for four squares from three grids collected by operator 4. The remaining holes are considered 'good'. Scale bars are 5 µm.

Figure S13: Analysis of holes from each square image for Operator 5

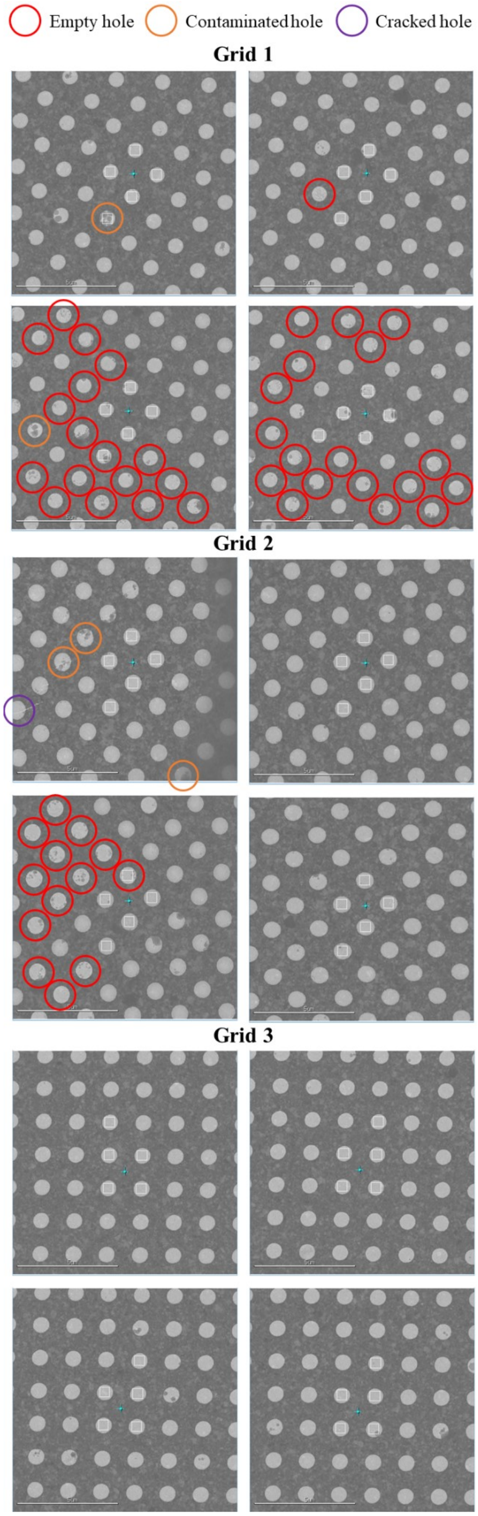

Figure S13 | Empty, contaminated, and cracked holes are identified for four squares from three grids collected by operator 5. The remaining holes are considered 'good'. Scale bars are 5 µm.

Figure S14: Analysis of holes from each square image for Smart Leginon Autoscreen

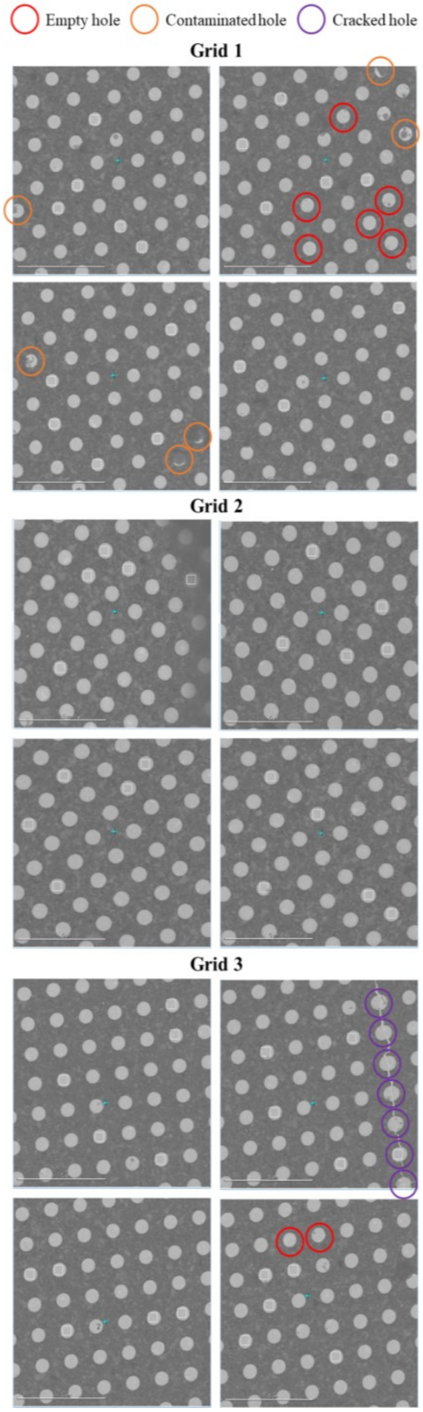

Figure S14 | Empty, contaminated, and cracked holes are identified for four squares from three grids collected by Smart Leginon Autoscreen. The remaining holes are considered 'good'. Scale bars are 5 µm.

Figure S15: Four user grids screened on a Krios with Smart Leginon before collection

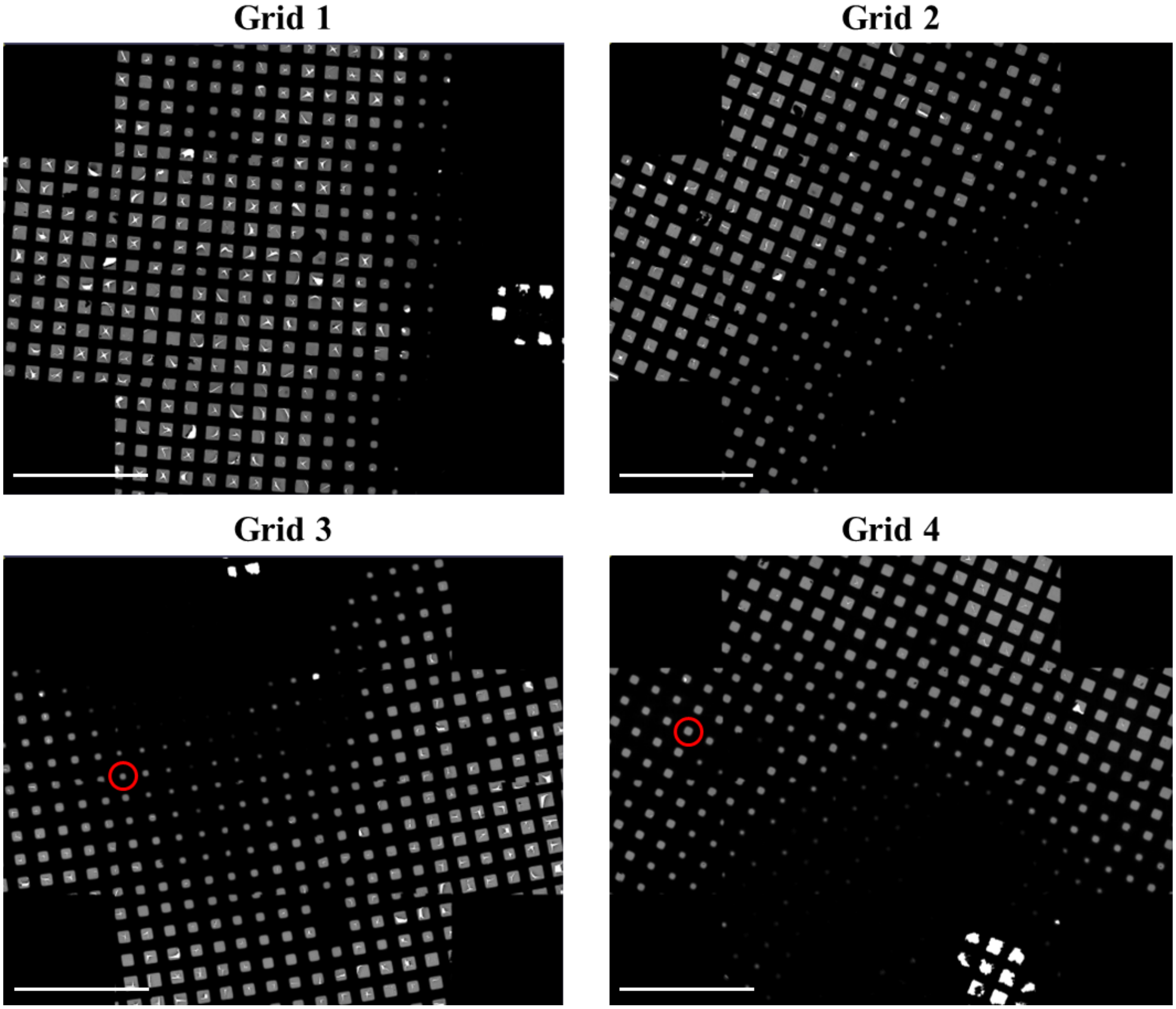

Figure S15 | Four user grids, ordered by the user from best to worst, were screened with Smart Leginon prior to setting up a 43-hour high-resolution collection. The 2-hour Smart Leginon MSI screening analysis determined that in fact the proper order from best to worst was Grid 4, Grid 3, then either Grid 2 or Grid 1. The squares circled in red on Grid 3 & 4 show example squares that were found to be optimal for collection. Several squares with different areas (Figure 5) needed to be screened on each grid before making this determination. Scale bars are 500 µm

Figure S16: Smart Leginon failure case at hole level

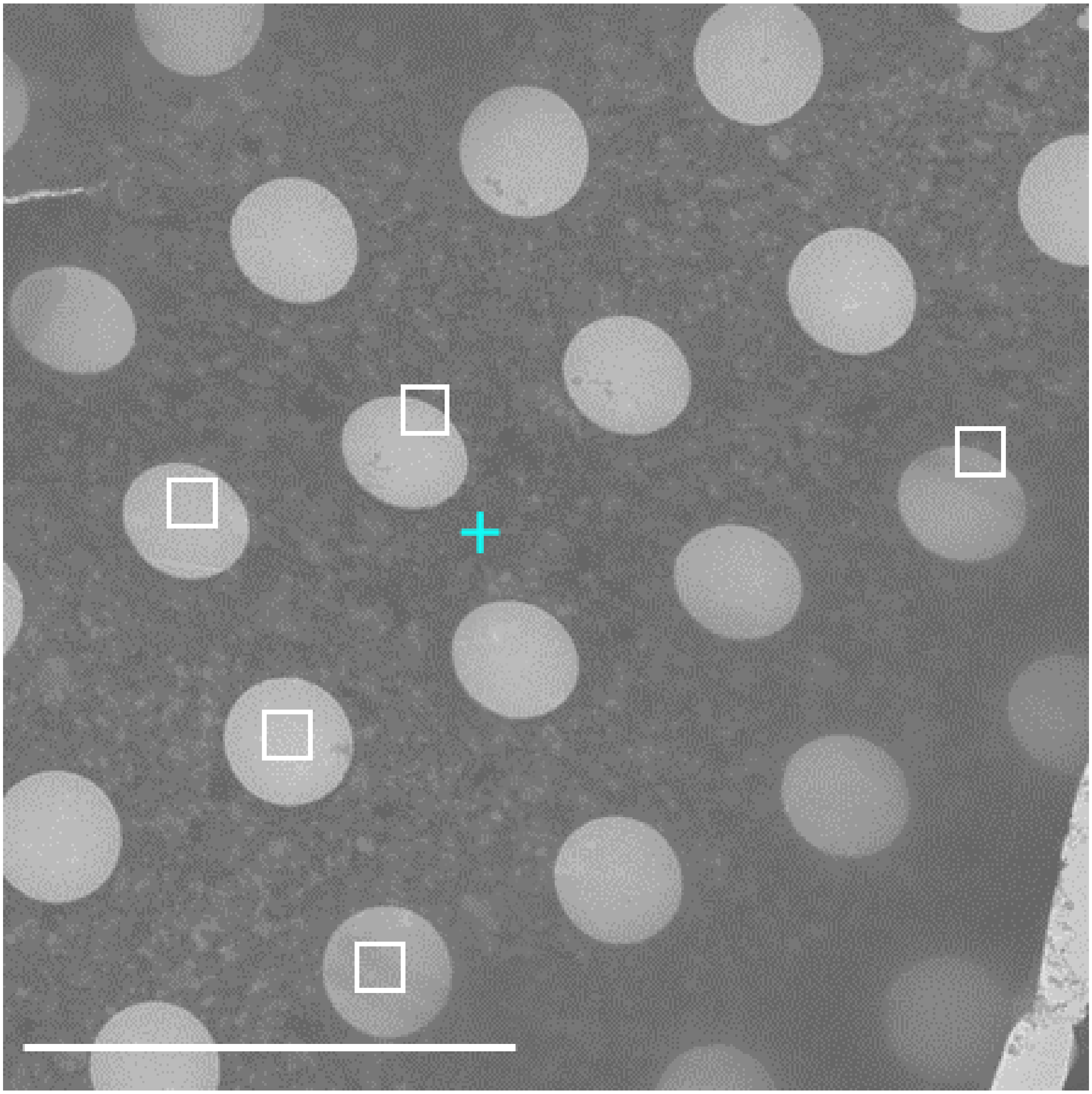

Figure S16 | A current limitation in Smart Leginon hole identification. Ptolemy hole lattice targeting on a grid with bent gold substrate shows that Ptolemy does not target well for non-square lattices. Scale bar is 5 µm.
